# miR-101, miR-548b, miR-554, and miR-1202 are reliable prognosis predictors of the miRNAs associated with cancer immunity in primary central nervous system lymphoma

**DOI:** 10.1371/journal.pone.0229577

**Published:** 2020-02-26

**Authors:** Yasuo Takashima, Atsushi Kawaguchi, Yasuo Iwadate, Hiroaki Hondoh, Junya Fukai, Koji Kajiwara, Azusa Hayano, Ryuya Yamanaka

**Affiliations:** 1 Laboratory of Molecular Target Therapy for Cancer, Graduate School for Medical Science, Kyoto Prefectural University of Medicine, Kyoto, Japan; 2 Center for Comprehensive Community Medicine, Faculty of Medicine, Saga University, Saga, Japan; 3 Department of Neurosurgery, Graduate School of Medical Sciences, Chiba University, Chiba, Japan; 4 Departments of Neurosurgery, Toyama Prefectural Central Hospital, Toyama, Japan; 5 Department of Neurological Surgery, Wakayama Medical University School of Medicine, Wakayama, Japan; 6 Department of Neurosurgery, Graduate School of Medical Sciences, Yamaguchi University, Ube, Yamaguchi, Japan; Hokkaido Daigaku, JAPAN

## Abstract

MicroRNAs (miRNAs) inhibit protein function by silencing the translation of target mRNAs. However, in primary central nervous system lymphoma (PCNSL), the expression and functions of miRNAs are inadequately known. Here, we examined the expression of 847 miRNAs in 40 PCNSL patients with a microarray and investigated for the miRNA predictors associated with cancer immunity-related genes such as T helper cell type 1/2 (Th-1/Th-2) and regulatory T cell (T-reg) status, and stimulatory and inhibitory checkpoint genes, for prognosis prediction in PCNSL. The aim of this study is to find promising prognosis markers based on the miRNA expression in PCNSL. We detected 334 miRNAs related to 66 cancer immunity-related genes in the microarray profiling. Variable importance measured by the random survival forest analysis and Cox proportional hazards regression model elucidated that 11 miRNAs successfully constitute the survival formulae dividing the Kaplan-Meier curve of the respective PCNSL subgroups. On the other hand, univariate analysis shortlisted 23 miRNAs for overall survival times, with four miRNAs clearly dividing the survival curves—miR-101/548b/554/1202. These miRNAs regulated Th-1/Th-2 status, T-reg cell status, and immune checkpoints. The miRNAs were also associated with gene ontology terms as Ras/MAP-kinase, ubiquitin ligase, PRC2 and acetylation, CDK, and phosphorylation, and several diseases including acquired immunodeficiency syndrome, glioma, and those related to blood and hippocampus with statistical significance. In conclusion, the results demonstrated that the four miRNAs comprising miR-101/548b/554/1202 associated with cancer immunity can be a useful prognostic marker in PCNSL and would help us understand target pathways for PCNSL treatments.

## Introduction

Primary central nervous system lymphoma (PCNSL) is a rare subgroup of diffuse large B-cell lymphoma (DLBCL), which is an aggressive variant of nodal non-Hodgkin lymphoma (NHL) [1]. PCNSL accounts for 3% of all primary central nervous system (CNS) tumors and 1% of NHLs observed in adults [2]. The 2016 WHO diagnostic criteria categorized most PCNSLs into an immune privileged site-associated DLBCL [1]. Despite intensive treatments with high-dose methotrexate (HD-MTX)-based polychemotherapy and deferred radiotherapy approach, the median overall survival (OS) of PCNSL patients shows poorer prognosis (approximately 4 years) than that of systemic DLBCL patients [3,4].

Recent studies in cancer immunity have advanced by targeting immune checkpoint molecules on cell surfaces which repress the responses of pro-inflammatory lymphocytes and cytotoxic T lymphocytes (CTLs) [5]. Checkpoint inhibitors as monoclonal antibodies block inhibitory checkpoint activation and then enhance T cell activities, thereby showing anticancer effects [6]. The T cell receptor responses of NHLs are augmented by monoclonal antibodies binding to the programmed cell death protein (PD-1/cluster of differentiation (CD) 279) and CTL-associated protein 4 (CTLA-4/CD152) [7–10]. Encouraging reports regarding efficacy of nivolumab in relapsed/refractory PCNSL were obtained in a small uncontrolled series (n = 4) [11]. A clinicopathological study revealed PD-L1 expression levels in tumor microenvironments compared to levels in tumor cells, demonstrated the correlation between the expression of CD4 and interferon-gamma (IFN-γ), and evaluated prognosis [12]. In another study on PD-1 ligands, signal transducer and activator of transcription 3 (STAT3) inhibitors evaded the expression of PD-1 ligands including CD274 (PD-L1) and CD273 (PD-L2) in a PCNSL-derived cell line, HKBML [13]. On the other hand, it has been shown that stimulus-dependent expression of PD-L1 and indoleamine 2, 3-dioxygenase 1 (IDO-1) by macrophage-interaction caused immune evasion of PCNSL-derived cell lines, HKBML and TK [14].

MicroRNAs (miRNAs) are small noncoding RNAs consisting of approximately 20–24 nucleotides, which inhibit translation and protein function of target mRNAs [15]. Various miRNAs are involved in cell cycle regulation, proliferation, differentiation, cell death, and cancer [16]. The mechanism of gene silencing by miRNAs is called RNA interference (RNAi) [17]. Dysregulation of RNAi is sometimes involved in tumor malignancy, as observed in chronic lymphocytic leukemia [18] or acute lymphoblastic leukemia [19]. Differential expression of miRNAs has been found in PCNSL and non-CNS DLBCL [20]. In PCNSL, miR-145, miR-193b, miR-199a, and miR-214 are reported to be downregulated [21]. On the other hand, miR-9, miR-17-5p, miR-20a, miR-30b/c, and miR-155 are observed to be upregulated and involved in the MYC pathway, terminal B-cell differentiation, and cytokine-dependent gene expression [22]. While several miRNAs are known as potential biomarkers in PCNSL [23–26], the biological function and clinical significance of these miRNAs have not yet been elucidated. As a result, the miRNAs required for gene regulation in cancer immunity have also largely been unknown in PCNSL therapeutic-based studies.

Here, we reveal a miRNA predictor through analysis of correlation between the miRNA expression and OS in 40 PCNSL patients. We particularly focused on the cancer immunity-related genes and the miRNAs targeting them. First, we screened 11 miRNA candidates from the 847 human miRNAs on microarray chips and performed multivariate Cox proportional hazards regression analysis for OS. Based on the results, we next constructed the survival prediction formulae dividing the Kaplan-Meier curves. Further, we limited the miRNA predictors into a low-dimensional formula by harboring only four miRNAs and investigated characteristics of these four miRNAs and their targets. The results demonstrated that the miRNA predictors can be used as a multi-marker prognostic tool and would help us understand the miRNA pathways for cancer immunity in PCNSL.

## Materials and methods

### Clinical specimens

Forty patients diagnosed with PCNSL were treated at Chiba University, Toyama Prefectural Central Hospital, Wakayama Medical University School of Medicine, and Yamaguchi University (S1 Table). The study was approved by The Ethics Committee of Kyoto Prefectural University of Medicine (RBMR-C-1082-1) and experiments were performed in accordance with the institutional guidelines. Written informed consent was obtained from all the patients. Statistical analysis for clinical information was performed using the JMP built-in modules (SAS Institute Inc., Tokyo, Japan).

### Microarray

Total RNA was extracted from approximately 100 mg of each tumor specimens using Isogen II (Nippongene, Toyama, Japan). The quality of the extracted RNA was verified using 2100 Bioanalyzer System with RNA Pico Chips (Agilent Technologies, Tokyo, Japan). Approximately 1 μg of RNA was amplified twice and hybridized on GeneChip miRNA 4.0 Array (Affymetrix Inc., Tokyo, Japan). Washing and staining of arrays, fluorescence detection, and image-quality analysis were performed using Fluidics Station 450, High-Resolution Microarray Scanner 3000, and GCOS Workstation Version 1.3, respectively (Affymetrix, Inc., Tokyo, Japan). The expression value of miRNAs was determined using Affymetrix Expression Console Software (Affymetrix, Inc., Tokyo, Japan). A part of the microarray data was deposited in the Gene Expression Omnibus (GEO) (GSE122011) [27]. Expression values were normalized by z-score.

### Quantitative polymerase chain reaction (qPCR)

RNA was reverse-transcribed using a Mir-X miRNA First-Strand Synthesis Kit (Takara Bio Inc., Shiga, Japan), as described [28]. Primers were used as follows: TAC AGT ACT GTG ATA ACT GAA (hsa-miR-101-3p), AAA AGT AAT TGT GGT TTT GGC C (hsa-miR-548b-5p), GCT AGT CCT GAC TCA GCC AGT (hsa-miR-554), and GTG CCA GCT GCA GTG GGG GAG (hsa-miR-1202). The values of U6 snRNA was used for normalization.

### Clustering analysis

Expression of miRNAs was clustered with Ward method using the JMP built-in modules (SAS Institute, Inc., Tokyo, Japan), as described [29].

### Random survival forests analysis

Random survival forests analysis was used to determine the variable importance factors distinguishing expression of miRNAs with microarray raw data, as described [30,31]. Briefly, the values of variable importance reflect the relative contribution of each variable to the prediction for the survival time. The variable importance was estimated by randomly permutating its values and recalculating the predictive accuracy of the model which were expressed as the log rank test statistics. The method was implemented by using the randomForestSRC package of the statistical software R.

### Cox proportional hazards analysis

The correlation between expression of the 334 miRNAs of interests and OS was evaluated by univariate and multivariate analyses. Clinical characteristics were used as an additional variable to perform multivariate analysis. The statistical data were determined with the Cox proportional hazards regression model using the JMP built-in modules (SAS Institute Inc., Tokyo, Japan), as described [30].

### Kaplan-Meier survival analysis

The Kaplan-Meier method was used to estimate the survival distributions for each subgroup with the log-rank test among subgroups using the JMP built-in modules (SAS Institute Inc, Tokyo, Japan.) [29].

### Receiver operating characteristic (ROC) analysis

Total forty subjects were randomly divided into training data and test data at a ratio of 3: 1. A regression equation was estimated for the training data using Cox regression, and a time-dependent ROC analysis was performed for the test data using the regression score. Area under the curve (AUC) was also calculated. The time of evaluation was two and five years survivals. This process was repeated 10,000 times, and the average value of AUC was calculated. The R package timeROC was used.

### Target prediction and gene ontology (GO) annotation

The study was limited on cancer immunity-related gene set slightly modified (66 genes, S2 Table) as described [32] and their targeted miRNAs (334 miRNAs, S3 Table). Cancer immunity-related genes were specifically categorized into four subgroups comprising T helper cell type 1 status (Th-1, 17 genes), Th-2 status (18 genes), regulatory T cell status (T-reg, 14 genes), and stimulatory (21 genes) and inhibitory (20 genes) checkpoints. The miRNAs targeting them were searched by TargetScanHuman 7.2. MicroRNAs were annotated using miRBase 22 (http://www.mirbase.org/) and their targets were surveyed using TargetScanHuman 7.2 (http://www.targetscan.org/vert_72/) with the TargetScan context++ scores (<-0.01) [33] on the Human GRCh38/hg38. Functional GO annotation was performed using miRBase and DAVID 6.8 (https://david.ncifcrf.gov/), as described [33].

### Statistics

Statistical analysis was performed using the JMP built-in modules (SAS Institute Inc., Tokyo, Japan). P < 0.05 was considered to be statistically significant.

## Results

### Clinical information of the patients with PCNSL

The aim of this study is to find promising prognosis markers based on the expression of miRNAs related to cancer immunity in PCNSL. This study was carried out on specimens from 40 PCNSL patients including training data set (n = 20) and test data set (n = 20) whose characteristics are described in S1 Table.

### Differential expression of cancer immunity miRNAs in PCNSL

**Fig 1:**
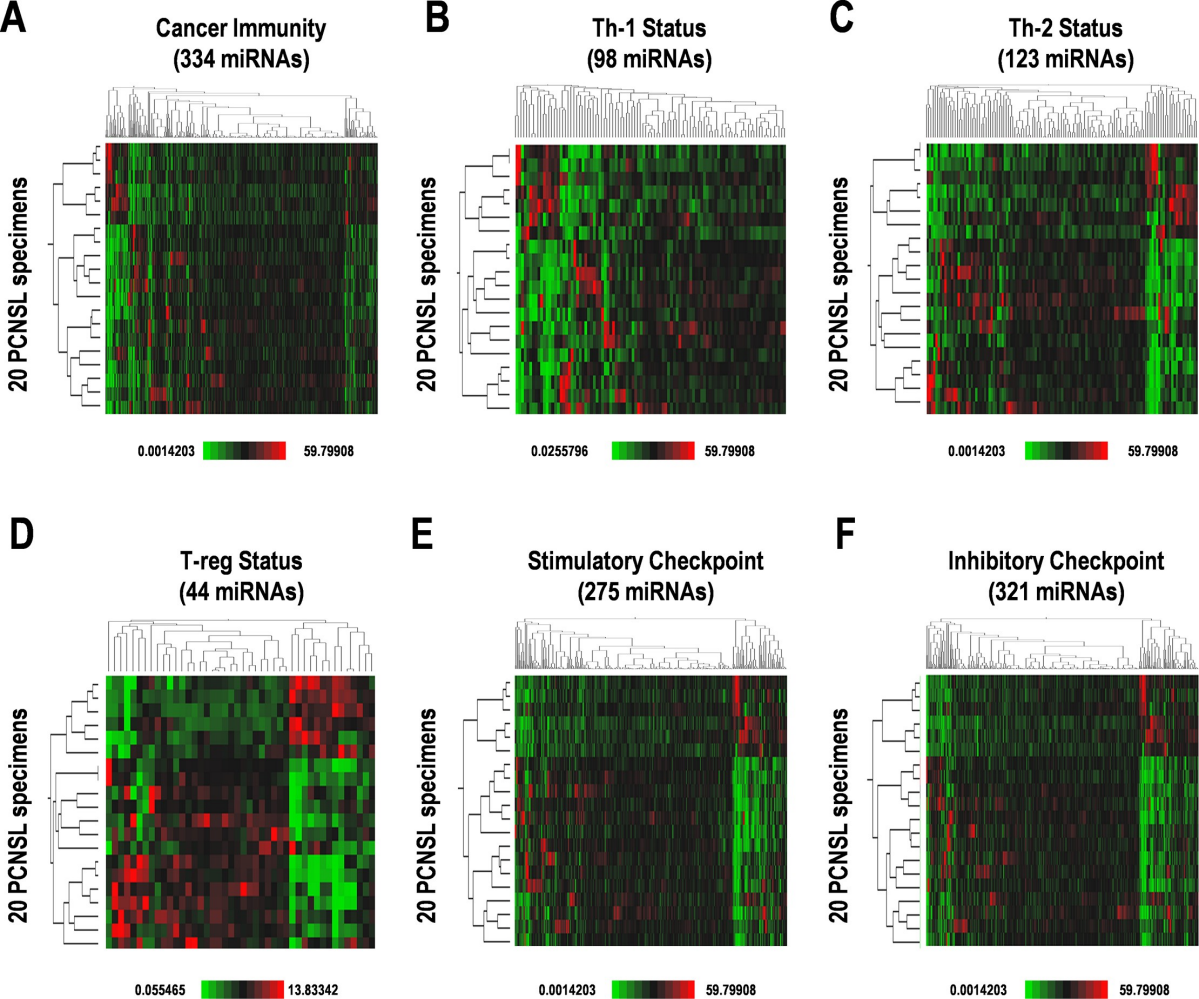
Differential expression of cancer immunity-related miRNAs in PCNSL patients. Hierarchical clustering of expression of miRNAs involved in (**A**) cancer immunity; and subgroups including (**B**) Th-1 status, (**C**) Th-2 status, (**D**) regulatory T-cell (T-reg) status, (**E**) stimulatory checkpoint, and (**F**) inhibitory checkpoint. Relatively high and low expression, designated as higher expression and lower expression by median expression, are indicated by red and green, respectively.

We performed a microarray for differential patterns of expression of miRNAs on the 40 PCNSL specimens and a total of 847 human miRNAs were detected (S2 Fig). Of the total miRNAs detected, 468 miRNAs were relatively highly-expressed and 197 miRNAs were detected as “high expression” in >50% specimens (S2 Fig). The 847 miRNAs included 334 miRNAs targeting “cancer immunity-related genes”, distributed as 98 miRNAs related to Th-1 status, 123 miRNAs for Th-2 status, 44 miRNAs for T-reg status, 275 miRNAs for stimulatory checkpoints, and 321 miRNAs for inhibitory checkpoints (Fig 1). Expression data were hierarchically clustered and divided into the two subgroups from the 20 PCNSL patients (Fig 1). However, Kaplan-Meier survival curves were not statistically divided into categories (S3 Fig). Therefore, these results suggest that appropriate combinations of miRNA expression are required to estimate prognosis in PCNSL.

### Variable importance of cancer immunity-related miRNAs in PCNSL

**Fig 2:**
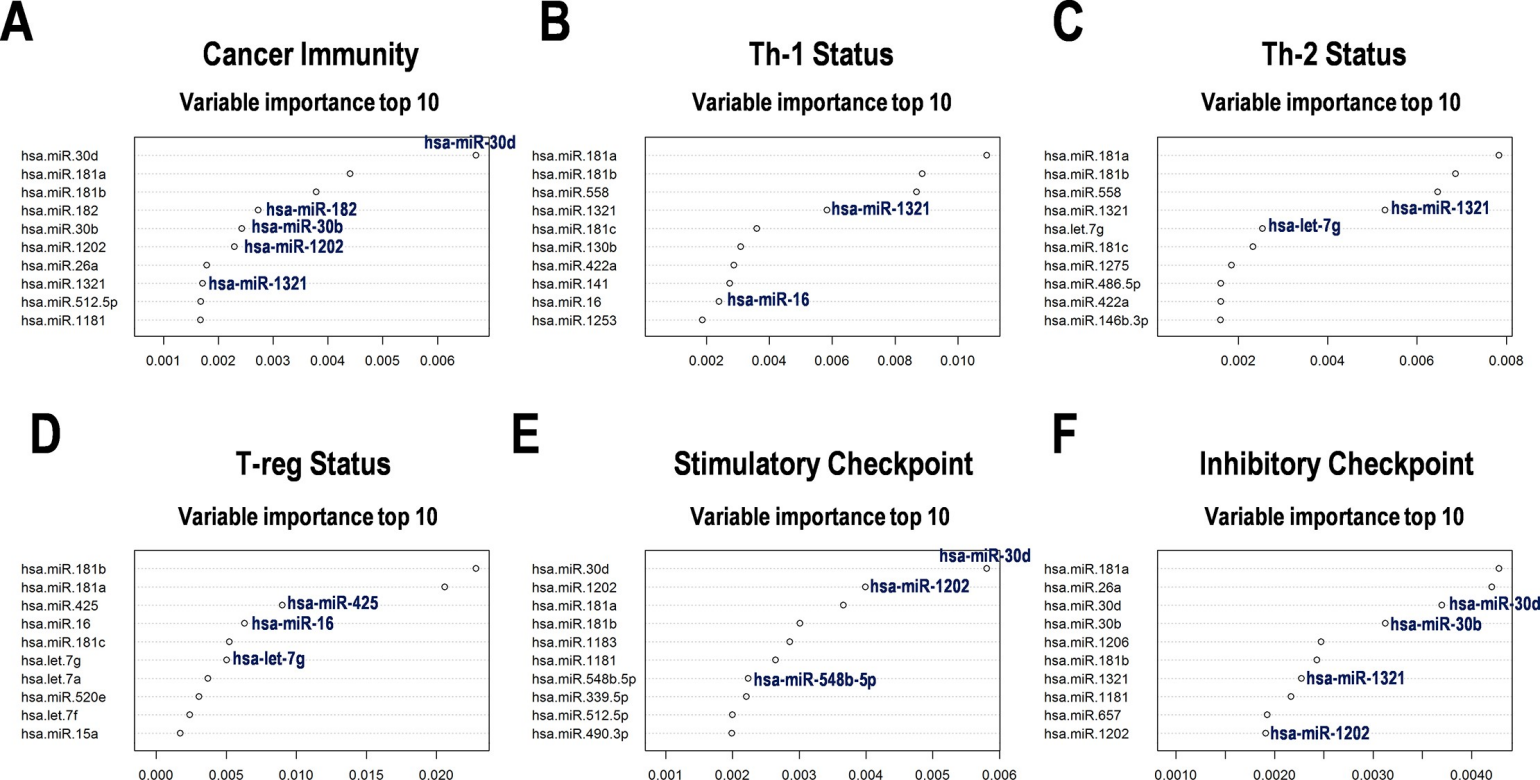
Variable importance of the cancer immunity-related miRNAs with the random survival forests analysis in the PCNSL patients. Variable importance of representative miRNAs involved in (**A**) cancer immunity; (**B**) subgroups including Th-1 status, (**C**)Th-2 status, (**D**) regulatory T-cell (T-reg) status, (**E**) stimulatory checkpoint, and (**F**) inhibitory checkpoint.

To further examine the contribution of miRNA expression to OS distribution of the PCNSL patients, we analyzed the data using a Random survival forests model (Fig 2). Variable importance score demonstrated a relatively strong appearance of miR-30b/d, miR-181a/b, miR-182, and miR-1202 in the total “cancer immunity-related genes” (Fig 2A); miR-181a/b, miR-558, and miR-1321 in “Th-1 status” and “Th-2 status” (Fig 2B and 2C); miR-16, miR-181a/b, and miR-425 in “T-reg status” (Fig 2D); miR-30d, miR-181a, and miR-1202 in “stimulatory checkpoint” (Fig 2E); and miR-26a, miR-30b/d, and miR-181a in “inhibitory checkpoint” (Fig 2F) groups. From the miRNAs associated with checkpoint gene regulation, these miRNAs were suggested to be predictive of survival in PCNSL.

### Cox regression analysis for survival times of the PCNSL patients

In addition, we evaluated the OS of PCNSL patients using a Cox proportional hazards model with expression of 334 cancer immunity-related miRNAs (S4 Table). The results indicated that the hazard ratio (HR) with statistical significance included miR-1202, miR-101, miR-372, and miR-609 in the total “cancer immunity-related genes” group, “stimulatory checkpoint”, and “inhibitory checkpoint” subgroups; miR-577, miR-454, and miR-920 in “Th-1 status”; miR-101 in “Th-2 status”; and miR-425, miR-16, and let-7g/i in “T-reg status” subgroups. Coupled with results from the Random survival forests analysis, it was evaluated that miR-1202 in the pathway of cancer immunity and immune checkpoints, and miR-16 and miR-425 involved in T-reg differentiation status are key factors for determining the OS of the PCNSL patients.

### Survival prediction formula with expression of cancer immunity-related miRNAs in PCNSL

**Fig 3:**
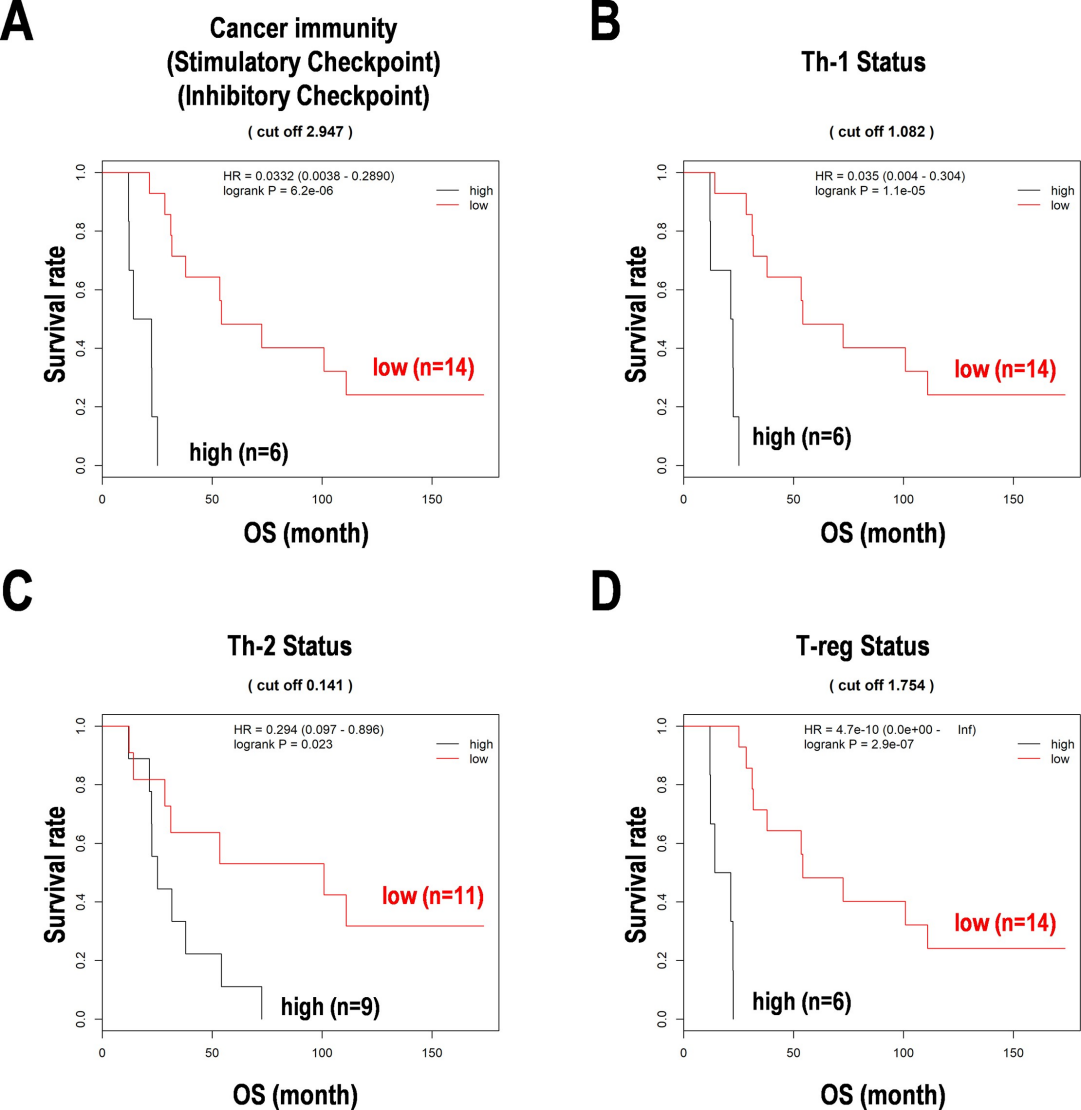
Survival distribution with the formulas based on the Cox regression analysis in PCNSL. (**A**) Cancer immunity and immune checkpoints (stimulatory and inhibitory checkpoints). (**B**) Th1 T-cell status. (**C**) Th2 T-cell status. (**D**) Regulatory T-cell (T-reg) status. The Kaplan-Meier curves are drawn by a cut off score calculated by each formula in PCNSL. OS, overall survival. HR, hazard ratio.

We constituted the survival prediction formulae by using the results from the Cox regression analysis (S4 Table). These formulae were used to divide the subgroups of the PCNSL patients (Fig 3). The Kaplan-Meier curves using the formulae with a cut off by a median score clearly divided the high-score and low-score subgroups with statistical significance in cancer immunity-related genes and immune checkpoint (Fig 3A), Th-1 status (Fig 3B), Th-2 status (Fig 3C), and T-reg status (Fig 3D). Each formula estimated higher score with poorer prognosis, suggesting the importance of expression patterns of the detected miRNA for cancer immunity in PCNSL patients of this study.

### The miRNA predictors for cancer immunity in PCNSL

**Fig 4:**
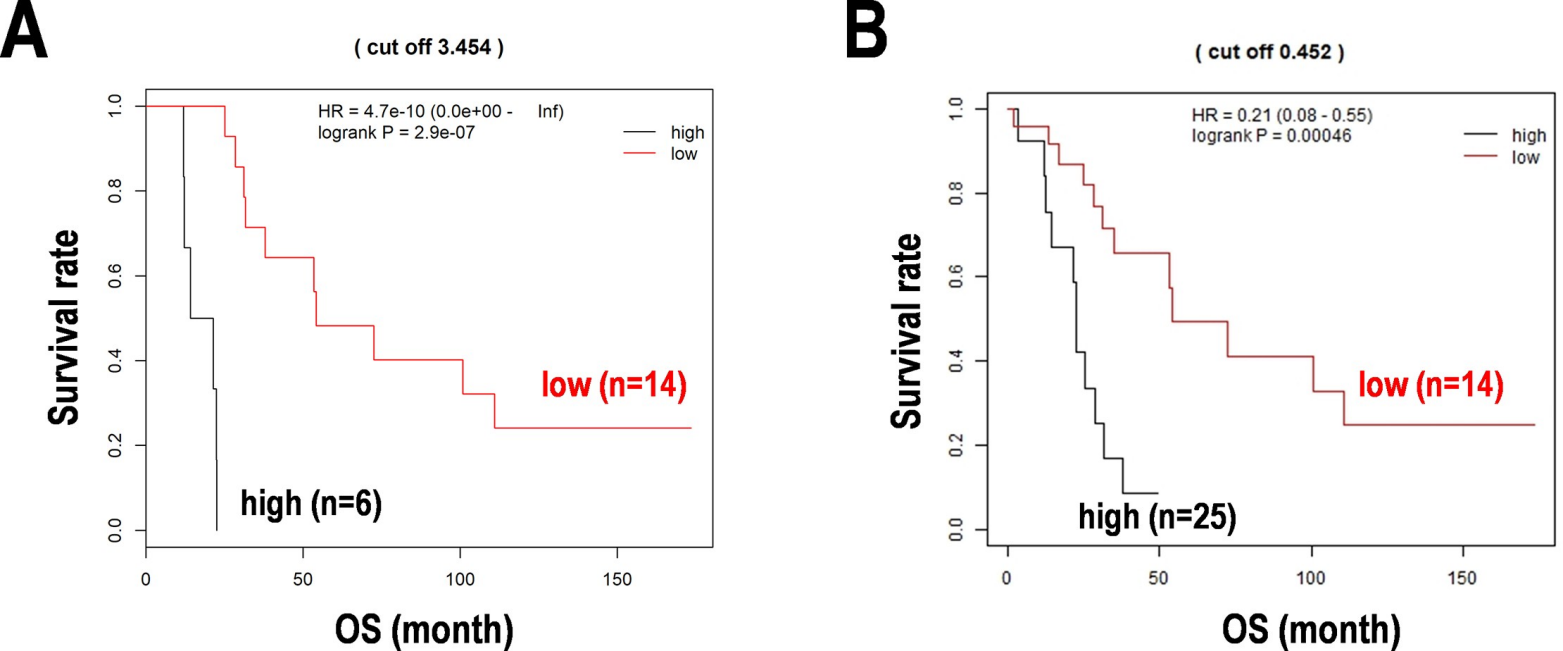
Re-evaluated prognosis prediction model for cancer immunity-related miRNAs in PCNSL. (**A**) Representative miRNAs encompassing cancer immunity in the training data set (n = 20). (**B**) Remodeling of the prognosis prediction for cancer immunity-related miRNAs with clinical information including age, gender, KPS, serum LDH level, and deep-seated lesion in the total data set (n = 39, one sample deleted due to a missing value of LDH). Kaplan-Meier analyses cut off by the median score calculated by the prognosis formula based on the Cox regression analysis. OS; overall survival, HR; hazard ratio.

The abovementioned 11 miRNAs, namely miR-16, miR-101, miR-372, miR-425, miR-454, miR-577, miR-609, miR-920, miR-1202, let-7g, and let-7i, were considered as miRNA predictors for cancer immunity in PCNSL. Further, we tried to limit the miRNA predictors by reanalyzing the study. As a result, 23 miRNAs including miR-1321, miR-30b/d, and miR-182 with identical variable importance from the Random survival forests analysis (Fig 2) and let-7g, miR-101, miR-1202, miR-577, miR-16, and miR-425 with a significant HR in univariable analyses from the Cox hazard model were collected (S5 Table). Then, the low-dimensional formula for prognosis prediction in PCNSL was constituted. The formula was used to divide the subgroups of PCNSL patients and the Kaplan-Meier curves with a cut off score clearly dividing the high-score and low-score subgroups with statistical significances (Fig 4A).

Furthermore, the formula was improved using representative four miRNAs, including miR-101, miR-548b-5p, miR-554, and miR-1202, with clinical information, including age, gender, Karnofsky performance status (KPS), serum lactate dehydrogenase (LDH) level (U/L), and deep-seated lesion, but the predictive formula was not influenced by the clinical status of the patients (S6 Table). Consequently, survival curves were successfully divided by the formula using the four miRNAs in the total data set (n = 39) (Fig 4B). Besides, the secondary training data set (n = 30) randomly divided from the total samples was also validated with Cox regression and time-dependent ROC analyses. In this internal validation, the ROC analysis returned the results in AUC 0.74 at 2-year survival and 0.91 at 5-year survival. The expression of the four miRNAs including miR-101 (r = 0.216), miR-548b-5p (r = 0.015), miR-554 (r = 0.138), and miR-1202 (r = 0.335) was also confirmed by qPCR (S4 Fig). These results were considered that miR-101, miR-548b-5p, miR-554, and miR-1202 constitute promising prognostic markers of the miRNAs associated with cancer immunity in PCNSL. However, whether these miRNAs are actually effective for cancer immunity should await future study.

### Possible roles of the miRNA predictors in PCNSL

miR-101, miR-548b-5p, miR-554, and miR-1202, the four miRNAs designated as miRNA predictors in PCNSL were widely associated with all categories such as Th-1/Th-2 and T-reg differentiation status, and stimulatory and inhibitory immune checkpoints (S7 Table). On the other hand, gene ontology (GO) terms generated from the target genes revealed potential roles in cell growth (e.g., vascular endothelial growth factor (VEGF), hepatocyte growth factor receptor (HGFR), mitogen-activated protein kinase (MAPK), Ras, guanosine diphosphate (GDP)-binding, guanosine triphosphate (GTP)ase, cyclin-dependent kinase (CDK), and signal transducer), transcription activity (e.g., forkhead box-containing protein, O sub-family (FoxO) signaling, nucleus, splicing, ubiquitin ligase, RNA-binding, acetylation, phosphoprotein, transcriptional misregulation, and pathways in cancer), and diseases (e.g., chronic myeloid leukemia, glioma, hippocampus, acquired immunodeficiency syndrome, blood-related disorders, angiogenesis, and heart defects) (S8 Table and S9 Table). These findings indicated that the miRNA predictors for cancer immunity in PCNSL was also associated with cell growth, stimulus-dependent signaling pathways, blood-related morphology, angiogenesis, immune diseases, CNS tumors including glioma, and metabolic disorders. This suggests the promising contribution of the miRNA predictors in understanding the cancer pathway and possible targeted therapy in PCNSL as well as in many other diseases.

## Discussion

In this study, we investigated miRNA predictors through correlation analysis between miRNA expression and OS using a Random survival forests model and a Cox hazard regression model in 40 PCNSL patients. We especially focused on the miRNAs targeting the cancer immunity-related genes. We performed a microarray and screened 11 potential miRNA candidates, namely miR-16, miR-101, miR-372, miR-425, miR-454, miR-577, miR-609, miR-920, miR-1202, let-7g, and let-7i, from a total of 847 human miRNAs detected. Based on the expression data, we constructed the prediction formula to divide Kaplan-Meier survival curves. Further, we limited the miRNA predictors into a low-dimensional formula by harboring only four miRNAs that included miR-101, miR-548b, miR-554, and miR-1202. miR-101 is associated with proliferation and apoptosis in DLBCL [34]. While, in glioblastoma, the miR-101 functions as a tumor suppressor targeting Krüppel-like factor (KLF)6 [35]. Although miR-548 has not been reported in CNS tumors including PCNSL, the miR-548 acts as an anti-oncogenic factor inhibiting the phosphoinositide 3-kinase (PI3K)/AKT signaling pathway in lung cancer [36] and associated with high-risk Gleason scores in prostate cancer [37]. The miR-548b inhibits cells proliferation and enhances apoptosis in breast cancer [38]. The miR-548b also inhibits proliferation and invasion in malignant glioma [39] and hepatocellular carcinoma (HCC) [40,41]. On the other hand, in esophageal squamous cell carcinoma (ESCC), the miR-548 enhances cell migration and invasion [42]. miR-1202 is a brain-specific miRNA in primates [43]. In glioma, the miR-1202 expression is inversely correlated with the expression of Ras-related protein Rab-1A (RAB1A) [44]. The miR-1202 also suppresses cell migration and invasion by targeting CDK14 in HCC [45]. The miR-1202 silencing increases apoptosis and G1-arrest while decreases migration and invasion in endometrial cancer *in vitro* [46].

In this study, these four miRNAs, including miR-101, miR-548b-5p, miR-554, and miR-1202, covered the total cancer immunity pathway (47/66 genes, 71.2%) comprising Th-1 status (12/17 genes, 70.5%), Th-2 status (12/18 genes, 66.6%), T-reg status (7/14 genes, 50%), stimulatory checkpoint (9/21 genes, 42.8%), and inhibitory checkpoint (7/20 genes, 35%) (S7 Table). It also suggested that a small number of miRNAs can efficiently regulate various pathways in cancer immunity. Besides, the four miRNAs were also predicted by GO to target the genes involved in cell growth, stimulus-dependent signaling, angiogenesis, blood morphology, immune diseases and tumors, and metabolic disorders. This suggested the potential role of the novel four miRNAs as co-factors in understanding other diseases. Random survival forests analysis on the miRNA expression profile in cancer immunity revealed identical variable importance in each category. Of these, some miRNAs (miR-30b/c, miR-26b, and let-7g) have already been reported as highly expressed biomarker candidates in PCNSL (not expressed in DLBCL) [21]. Increased expression of miR-30c in secondary CNS lymphoma patients allows the lymphomas to engraft into the CNS by suppressing the cadherin EGF LAG seven-pass G-type receptor (CELSR)3 gene that encodes the flamingo cadherin subfamily [47]. Therefore, the miR-30c is considered as a biomarker to distinguish PCNSL from secondary CNS lymphoma. The upregulation of miR-30d is also found in Hodgkin lymphoma [48]. Besides, differentially expressed miR-30d is reported as a reliable biomarker candidate in CNS-DLBCL [49]. A previous study has reported the downregulation of let-7e in DLBCL compared to normal lymph nodes whereas the reduced expression of let-7g shows a good event-free survival [50]. The let-7 family, which is repressed maturation by LIN28 family [28], targets the Ras/MAP-Kinase pathway [51]. Serum biomarkers have revealed highly expressed miR-16 in DLBCL [52].

Although our data are limited, the resultant four miRNAs including, including miR-101, miR-548b-5p, miR-554, and miR-1202, appear to efficiently control the cancer immunity-related genes. However, a recent study reported that intrinsic and extrinsic factors in T cells play a role in cancer immunity, such as adaptive immune and acquired resistance, against checkpoint inhibitors [53]. If so, we should create and develop innovative methods as an alternative to conventional immunotherapy with a checkpoint blockade using monoclonal antibodies such as cell-based reprogramming of cancer cells [54]. MiRNA approaches via RNAi pathways might be then available to repress checkpoint molecules in cancer-cell reprogramming-based immunotherapy.

## Supporting information

S1 Fig Survival distribution in PCNSL. (**A**) 20 PCNSL patients. (**B**) Based on gender. (**C**) Based on diagnosis age of 60. (**D**) Treatments by high dose-methotrexate (HD-MTX) or polychemotherapy. The Kaplan-Meier analysis was used to estimate overall survival (OS) time of PCNSL patients.
(PDF)

S2 Fig Expression ratio of miRNAs detected with the microarray in PCNSL. 468 miRNAs were detected in the 20 PCNSL specimens. 197 miRNAs were detectable in more than 50% of PCNSL specimens. The expression level over and under the median expression of each miRNA is referred as “expressed” and “not expressed”, respectively.
(PDF)

S3 Fig Kaplan-Meier analysis based on the hierarchical cluster method for the expression of miRNAs in 20 PCNSL patients. (**A**) Cancer immunity and immune checkpoint (stimulatory and inhibitory checkpoints). (**B**) Th-1 or Th-2 helper T-cell status. (**C**) T-reg status. HR; hazard ratio, OS; overall survival.
(PDF)

S4 Fig The correlation of the expression of the representative miRNAs between the microarray and the qPCR. (**A**) hsa-miR-101, (**B**) hsa-miR-1202, (**C**) hsa-miR-548b, and (**D**) hsa-miR-554. Scatter plots were shown with statistic results. Blue dot represents a value in the PCNSL specimen. Dotted lines indicate regression lines with correlation coefficient (r).
(PDF)

S1 Table Statistics of patients with PCNSL. (PDF)

S2 Table The cancer immunity-related genes. (PDF)

S3 Table The miRNA-binding candidates to the cancer immunity-related genes. (PDF)

S4 Table Cox hazard regression analysis for the cancer immunity-related miRNAs in PCNSL. (PDF)

S5 Table Significant miRNA candidates in cancer immunity in PCNSL. (PDF)

S6 Table Re-evaluated miRNAs with clinical information in PCNSL. (PDF)

S7 Table Target candidates of the miRNA predictors for cancer immunity in PCNSL. (PDF)

S8 Table Characterization of the target candidates of the miRNA predictors for cancer immunity in PCNSL. (PDF)

S9 Table Related diseases of the target candidates of the miRNA predictors for cancer immunity in PCNSL. (PDF)
