# Supplementary material for: miR-101, miR-548b, miR-554, and miR-1202 are reliable prognosis predictors of the miRNAs associated with cancer immunity in primary central nervous system lymphoma
Source: PLoS One. 2020 Feb 26;15(2):e0229577. doi: 10.1371/journal.pone.0229577 (PMC7043771; doi:10.1371/journal.pone.0229577)
Supplement: S1 Fig — (A) 20 PCNSL patients. (B) Based on gender. (C) Based on diagnosis age of 60. (D) Treatments by high dose-methotrexate (HD-MTX) or polychemotherapy. The Kaplan-Meier analysis was used to estimate overall survival (OS) time of PCNSL patients. (PDF) [file pone.0229577.s001.pdf]

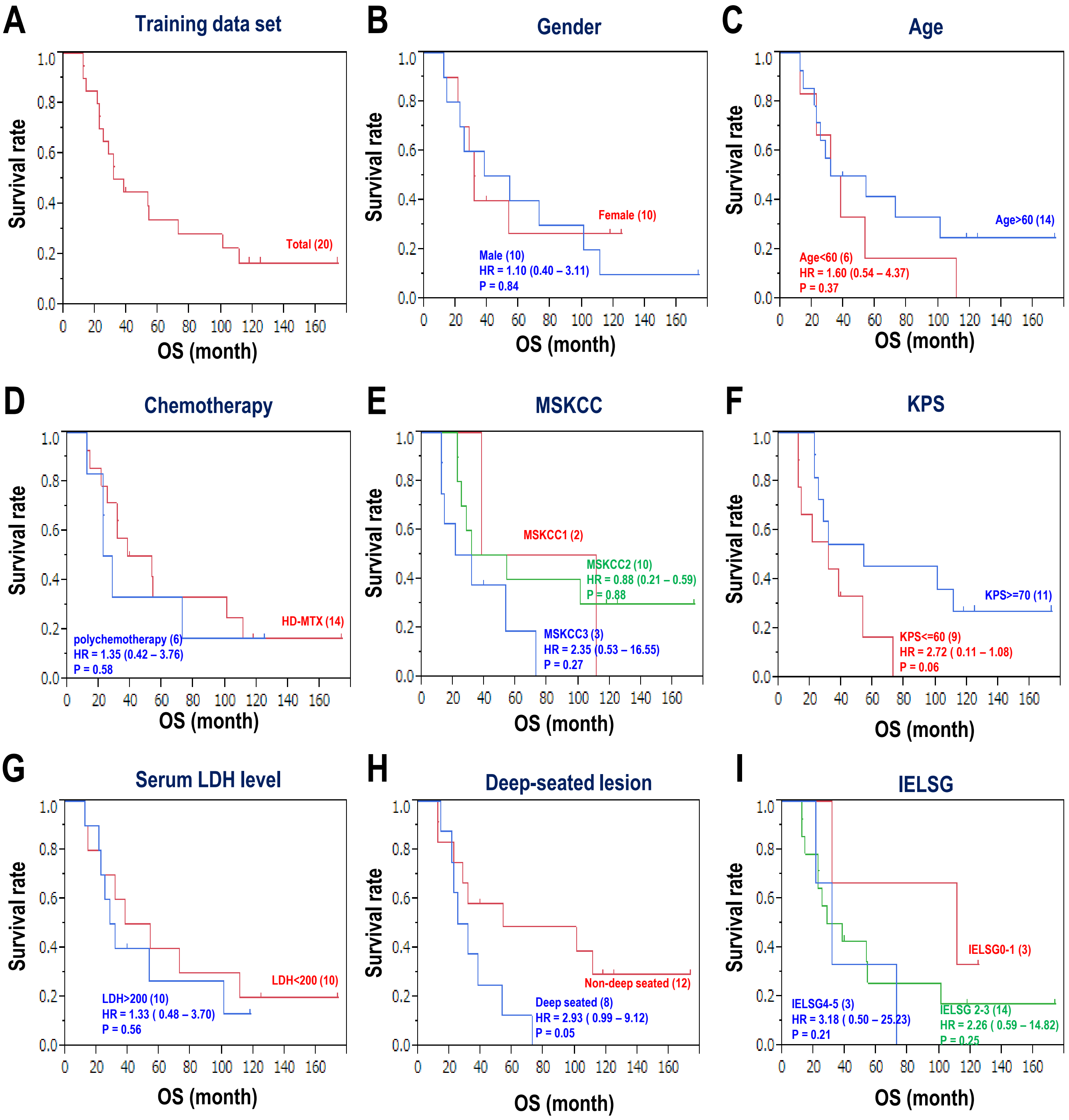

S1 Fig. Survival distribution in PCNSL. (**A**) Total samples in the training data set. (**B**) Gender. (**C**) Diagnosis age. (**D**) Chemotherapy by HD-MTX or polychemotherapy. (**E**) MSKCC score. (**F**) KPS. (**G**) Serum LDH level. (**H**) Deep-seated lesion. (**I**) IELSG. HR; hazard ratio, OS; overall survival.
