# Supplementary material for: miR-101, miR-548b, miR-554, and miR-1202 are reliable prognosis predictors of the miRNAs associated with cancer immunity in primary central nervous system lymphoma
Source: PLoS One. 2020 Feb 26;15(2):e0229577. doi: 10.1371/journal.pone.0229577 (PMC7043771; doi:10.1371/journal.pone.0229577)
Supplement: S2 Fig — 468 miRNAs were detected in the 20 PCNSL specimens. 197 miRNAs were detectable in more than 50% of PCNSL specimens. The expression level over and under the median expression of each miRNA is referred as “expressed” and “not expressed”, respectively. (PDF) [file pone.0229577.s002.pdf]

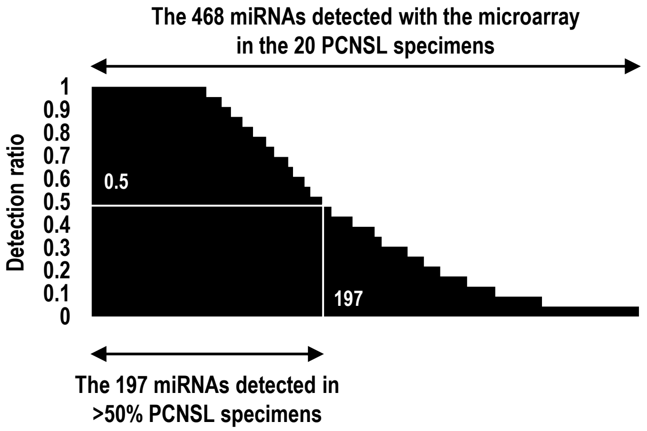

S2 Fig. Expression ratio of miRNAs detected with the microarray in PCNSL. 468 miRNAs were detected in the 20 PCNSL specimens. 197 miRNAs were detectable in more than 50% of PCNSL specimens. The expression level over and under the median expression of each miRNA is referred as “expressed” and “not expressed”, respectively.
