# Supplementary material for: miR-101, miR-548b, miR-554, and miR-1202 are reliable prognosis predictors of the miRNAs associated with cancer immunity in primary central nervous system lymphoma
Source: PLoS One. 2020 Feb 26;15(2):e0229577. doi: 10.1371/journal.pone.0229577 (PMC7043771; doi:10.1371/journal.pone.0229577)
Supplement: S3 Fig — (A) Cancer immunity and immune checkpoint (stimulatory and inhibitory checkpoints). (B) Th-1 or Th-2 helper T-cell status. (C) T-reg status. HR; hazard ratio, OS; overall survival. (PDF) [file pone.0229577.s003.pdf]

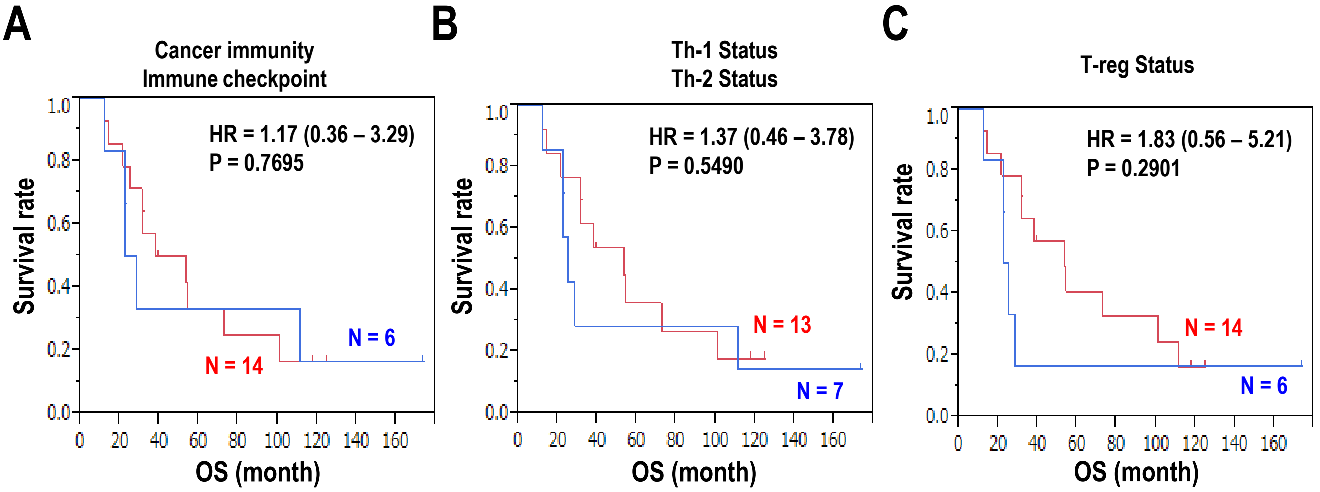

S3 Fig. Kaplan-Meier analysis based on the hierarchical cluster method for the expression of miRNAs in 20 PCNSL patients. (**A**) Cancer immunity and immune checkpoint (stimulatory and inhibitory checkpoints). (**B**) Th-1 or Th-2 helper T-cell status. (**C**) T-reg status. HR; hazard ratio, overall survival; OS.
