# Supplementary material for: miR-101, miR-548b, miR-554, and miR-1202 are reliable prognosis predictors of the miRNAs associated with cancer immunity in primary central nervous system lymphoma
Source: PLoS One. 2020 Feb 26;15(2):e0229577. doi: 10.1371/journal.pone.0229577 (PMC7043771; doi:10.1371/journal.pone.0229577)
Supplement: S4 Fig — (A) hsa-miR-101, (B) hsa-miR-1202, (C) hsa-miR-548b, and (D) hsa-miR-554. Scatter plots were shown with statistic results. Blue dot represents a value in the PCNSL specimen. Dotted lines indicate regression lines with correlation coefficient (r). (PDF) [file pone.0229577.s004.pdf]

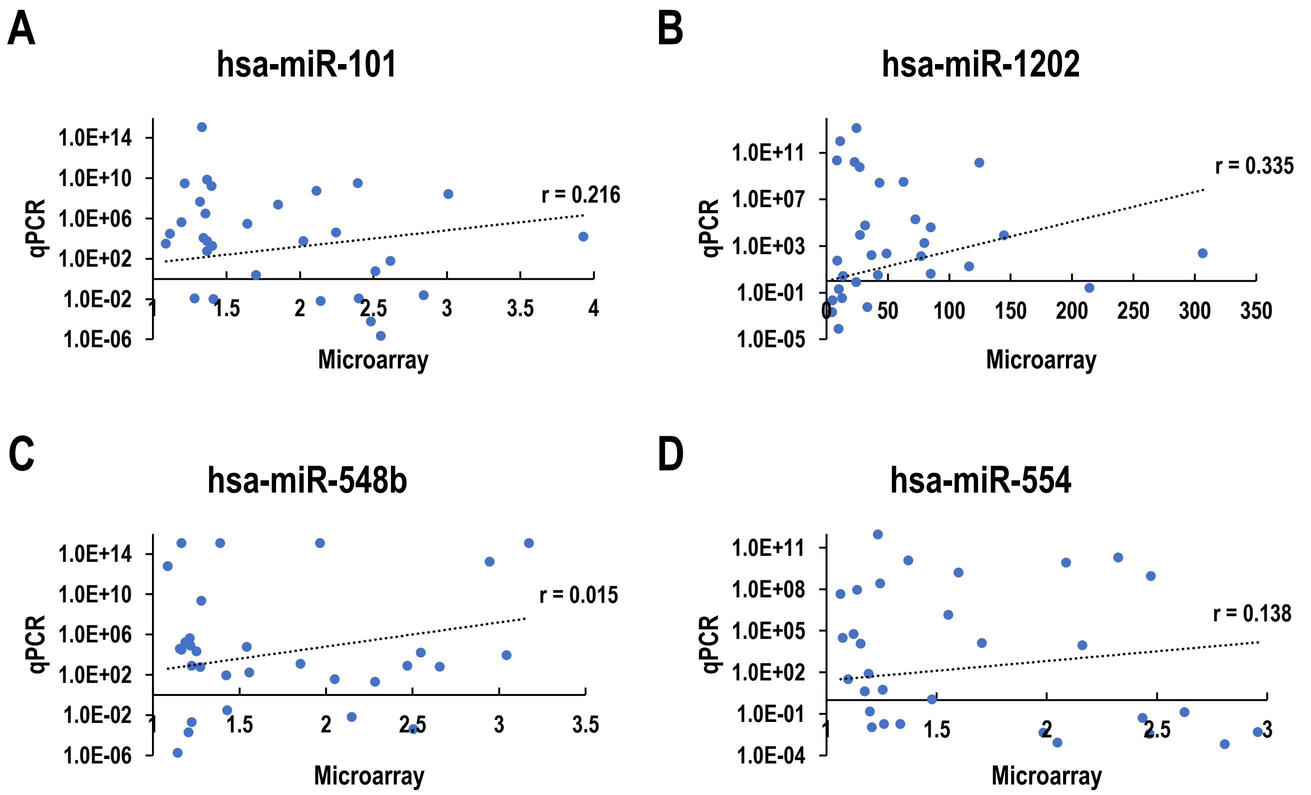

S4 Fig. The correlation of the expression of the representative miRNAs between the microarray and the qPCR. (**A**) hsa-miR-101, (**B**) hsa-miR-1202, (**C**) hsa-miR-548b, and (**D**) hsa-miR-554. Scatter plots were shown with statistic results. Blue dot represents a value in the PCNSL specimen. Dotted lines indicate regression lines with correlation coefficient (r).
