# Supplementary material for: miR-101, miR-548b, miR-554, and miR-1202 are reliable prognosis predictors of the miRNAs associated with cancer immunity in primary central nervous system lymphoma
Source: PLoS One. 2020 Feb 26;15(2):e0229577. doi: 10.1371/journal.pone.0229577 (PMC7043771; doi:10.1371/journal.pone.0229577)
Supplement: S1 Table — (PDF) [file pone.0229577.s005.pdf]

S1 Table. Statistics of patients with PCNSL.

		Training data set	Test data set
number (%)		20 (100)	20 (100)
Age (year-old)	average	64.05	65.2
	median	64.5	69
	min	44	23
	max	76	86
Age>60		14 (70)	14 (70)
Age<60		6 (30)	6 (30)
Gender	Female	10 (50)	7 (35)
	Male	10 (50)	13 (65)
OS[1] (months)	average	55.31	22.14
	median	34.85	21.2
	min	12	2
	max	173.5	49.5
chemotherapy	HD-MTX[2]	14 (70)	15 (75)
	polychemotherapy	6 (30)	2 (10)
MSKCC[3]	MSKCC1	2 (10)	3 (15)
	MSKCC2	10 (50)	5 (25)
	MSKCC3	3 (15)	12 (60)
KPS[4]	average	68	63.5
	median	70	60
	min	40	40
	max	100	100
KPS>=70		11 (55)	7 (35)
KPS<=60		9 (45)	13 (65)
Serum LDH[5] (U/L)	average	221.65	245.68
	median	199	216
	min	152	146
	max	407	767
LDH>=200		10 (50)	12 (60)
LDH<200		10 (50)	7 (35)
Deep seated lesion	Deep seated	8 (40)	13 (65)
	Non-deep seated	12 (60)	7 (35)
IELSG[6]	IELSG0-1	3 (15)	3 (15)
	IELSG2-3	14 (70)	10 (50)
	IELSG4-5	3 (15)	7 (35)

Note: [1]OS; overall survival, [2]HD-MTX; high-dose methotrexate, [3]MSKCC, Memorial Sloan-Kettering Cancer Center, [4]KPS; Karnofsky performance status, [5]LDH; lactate dehydrogenase, [6]IELSG; International Extranodal Lymphoma Study Group.
