# Supplementary material for: miR-101, miR-548b, miR-554, and miR-1202 are reliable prognosis predictors of the miRNAs associated with cancer immunity in primary central nervous system lymphoma
Source: PLoS One. 2020 Feb 26;15(2):e0229577. doi: 10.1371/journal.pone.0229577 (PMC7043771; doi:10.1371/journal.pone.0229577)
Supplement: S2 Table — (PDF) [file pone.0229577.s006.pdf]

S2 Table. The cancer immunity-related genes.

Symbol	GeneBank	Description	Alias name	Th-1	Th-2	T-reg	Stimulatory	Inhibitory
CD28	NM_006139	CD28 molecule	Tp44	CD28	CD28		CD28	
CD3D	NM_000732	CD3d molecule	CD3-DELTA, IMD19, T3D	CD3D	CD3D			
CD3E	NM_000733	CD3e molecule	IMD18, T3E, TCRE	CD3E	CD3E			
CD3G	NM_000073	CD3g molecule	CD3-GAMMA, IMD17, T3G	CD3G	CD3G			
CD4	NM_000616	CD4 molecule	CD4mut	CD4	CD4	CD4		
CD40LG	NM_000074	CD40 ligand	CD154, CD40L, HIGM1, IGM, IMD3, T-BAM, TNFSF5, TRAP, gp39, hCD40L	CD40LG	CD40LG		CD40LG	
CSF2	NM_000758	colony stimulating factor 2	GMCSF	CSF2	CSF2			
IFNG	NM_000619	interferon gamma	IFG, IFI	IFNG		IFNG		
IL12RB2	NM_001559	interleukin 12 receptor subunit beta 2		IL12RB2				
IL18R1	NM_003855	interleukin 18 receptor 1	CD218a, CDw218a, IL-1Rrp, IL18RA, IL1RRP	IL18R1	IL18R1			
IL2	NM_000586	interleukin 2	IL-2, TCGF, lymphokine	IL2				
IL3	NM_000588	interleukin 3	IL-3, MCGF, MULTI-CSF	IL3	IL3			
LTA	NM_000595	lymphotoxin alpha	LT, TNFB, TNFSF1	LTA				
STAT1	NM_007315	signal transducer and activator of transcription 1	CANDF7, IMD31A, IMD31B, IMD31C, ISGF-3, STAT91	STAT1				
STAT4	NM_003151	signal transducer and activator of transcription 4	SLEB11	STAT4				
TBX21	NM_013351	T-box 21	T-PET, T-bet, TBET, TBLYM	TBX21				
TNF	NM_000594	tumor necrosis factor	DIF, TNF-alpha, TNFA, TNFSF2	TNF				
GATA3	NM_002051	GATA binding protein 3	HDR, HDRS		GATA3			
IL10	NM_000572	interleukin 10	CSIF, GVHDS, IL-10, IL10A, TGIF		IL10	IL10		
IL13	NM_002188	interleukin 13	IL-13, P600		IL13			
IL4	NM_000589	interleukin 4	BCGF-1, BCGF1, BSF-1, BSF1, IL-4		IL4	IL4		
IL5	NM_000879	interleukin 5	EDF, IL-5, TRF		IL5			
IL6	NM_000600	interleukin 6	BSF2, HGF, HSF, IFNB2, IL-6		IL6			
IL9	NM_000590	interleukin 9	HP40, IL-9, P40		IL9			
STAT6	NM_003153	signal transducer and activator of transcription 6	D12S1644, IL-4-STAT, STAT6B, STAT6C		STAT6			
TGFB3	NM_003239	transforming growth factor beta 3	ARVD, ARVD1, RNHF, TGF-beta3, Transforming growth factor, beta 3, LDS5		TGFB3	TGFB3		
CD163	NM_004244	CD163 molecule	CD163, M130, MM130, SCARI1, CD163 molecule			CD163		
CD274	NM_014143	CD274 molecule	B7-H, B7H1, PD-L1, PDCD1L1, PDCD1LG1, PDL1			CD274		CD274
CD8A	NM_171827	CD8a molecule	CD8A, CD8, Leu2, MAL, p32, CD8a molecule			CD8A		
CTLA4	NM_005214	cytotoxic T-lymphocyte associated protein 4	ALPS5, CD, CD152, CELIAC3, CTLA-4, GRD4, GSE, IDDM12			CTLA4		CTLA4
FOXP3	NM_014009	forkhead box P3	FOXP3, AIID, DIETER, IPEX, JM2, PIDX, XPID, forkhead box P3			FOXP3		
PDCD1	NM_005018	programmed cell death 1	CD279, PD-1, PD1, SLEB2, hPD-1, hPD-l, hSLE1			PDCD1		PDCD1
PDCD1LG2	NM_025239	programmed cell death 1 ligand 2	B7DC, Btdc, CD273, PD-L2, PDCD1L2, PDL2, programmed cell death 1 ligand 2			PDCD1LG2		PDCD1LG2
TGFB1	NM_000660	transforming growth factor beta 1	CED, DPD1, LAP, TGFB, TGFbeta, transforming growth factor beta 1			TGFB1		
TGFB2	NM_003238	transforming growth factor beta 2	LDS4, TGF-beta2, transforming growth factor beta 2, G-TSF			TGFB2		
CD226	NM_006566	CD226 molecule					CD226	
CD27	NM_001242	CD27 molecule	S152, S152. LPFS2, T14, TNFRSF7, Tp55				CD27	
CD276	NM_025240	CD276 molecule	4Ig-B7-H3, B7-H3, B7H3, B7RP-2				CD276	CD276
CD40	NM_152854	CD40 molecule					CD40	
CD70	NM_001252	CD70 molecule					CD70	
CD80	NM_005191	CD80 molecule					CD80	CD80
HHLA2	NM_007072	HERV-H LTR-associating 2					HHLA2	
ICOS	NM_012092	inducible T-cell costimulator	AILIM, CD278, CVID1				ICOS	
IL2RB	NM_000878	interleukin 2 receptor subunit beta	CD122, IL15RB, P70-75				IL2RB	
PVR	NM_006505	poliovirus receptor					PVR	PVR
TMIGD2	NM_144615	transmembrane and immunoglobulin domain containing 2					TMIGD2	
TNFRSF14	NM_003820	TNF receptor superfamily member 14					TNFRSF14	TNFRSF14
TNFRSF18	NM_004195	TNF receptor superfamily member 18	AITR, CD357, GITR, GITR-D				TNFRSF18	
TNFRSF4	NM_003327	TNF receptor superfamily member 4					TNFRSF4	
TNFRSF9	NM_001561	TNF receptor superfamily member 9	4-1BB, CD137, CDw137, ILA				TNFRSF9	
TNFSF14	NM_003807	tumor necrosis factor superfamily member 14					TNFSF14	
TNFSF18	NM_005092	tumor necrosis factor superfamily member 18					TNFSF18	
TNFSF4	NM_003326	tumor necrosis factor superfamily member 4	CD134L, CD252, GP34, OX-40L, OX4OL, TXGP1				TNFSF4	
TNFSF9	NM_003811	tumor necrosis factor superfamily member 9					TNFSF9	
BTLA	NM_181780	B and T lymphocyte associated	BTLA1, CD272					BTLA
CD160	NM_007053	CD160 molecule						CD160
CD86	NM_175862	CD86 molecule						CD86
CD96	NM_198196	CD96 molecule						CD96
CEACAM1	NM_001712	carcinoembryonic antigen related cell adhesion molecule 1						CEACAM1
HAVCR2	NM_032782	hepatitis A virus cellular receptor 2	CD366, HAVcr-2, KIM-3, TIM3, TIMD-3, TIMD3, Tim-3					HAVCR2
IDO1	NM_002164	indoleamine 2,3-dioxygenase 1	IDO, IDO-1, INDO					IDO1
LAG3	NM_002286	lymphocyte activating 3	CD223					LAG3
LGALS3	NM_002306	galectin 3						LGALS3
LGALS9	NM_002308	galectin 9						LGALS9
TIGIT	NM_173799	T-cell immunoreceptor with Ig and ITIM domains						TIGIT
VTCN1	NM_024626	V-set domain containing T cell activation inhibitor 1						VTCN1
