# Supplementary material for: miR-101, miR-548b, miR-554, and miR-1202 are reliable prognosis predictors of the miRNAs associated with cancer immunity in primary central nervous system lymphoma
Source: PLoS One. 2020 Feb 26;15(2):e0229577. doi: 10.1371/journal.pone.0229577 (PMC7043771; doi:10.1371/journal.pone.0229577)
Supplement: S3 Table — (PDF) [file pone.0229577.s007.pdf]

S3 Table. The miRNA-binding candidates to the cancer immunity-related genes.

miRNA	Target gene	Position of target region	Predicted target sequence	Site type	Context++ score	Context++ score percentile	Weighted context++ score	Conserved branch length	P_{CT}
hsa-let-7d-5p	TNFSF9	Position 92-99 of TNFSF9 3' UTR	5'-UCCCUGCUGCUUUCUCUACCUCA	8mer	-0.67	99	-0.67	5.414	0.95
hsa-miR-4458	TNFSF9	Position 92-99 of TNFSF9 3' UTR	5'-UCCCUGCUGCUUUCUCUACCUCA	8mer	-0.66	99	-0.66	5.414	0.95
hsa-let-7f-5p	TNFSF9	Position 92-99 of TNFSF9 3' UTR	5'-UCCCUGCUGCUUUCUCUACCUCA	8mer	-0.64	99	-0.64	5.414	0.95
hsa-let-7g-5p	TNFSF9	Position 92-99 of TNFSF9 3' UTR	5'-UCCCUGCUGCUUUCUCUACCUCA	8mer	-0.64	99	-0.64	5.414	0.95
hsa-miR-98-5p	TNFSF9	Position 92-99 of TNFSF9 3' UTR	5'-UCCCUGCUGCUUUCUCUACCUCA	8mer	-0.64	99	-0.64	5.414	0.95
hsa-let-7a-5p	TNFSF9	Position 92-99 of TNFSF9 3' UTR	5'-UCCCUGCUGCUUUCUCUACCUCA	8mer	-0.63	99	-0.63	5.414	0.95
hsa-let-7b-5p	TNFSF9	Position 92-99 of TNFSF9 3' UTR	5'-UCCCUGCUGCUUUCUCUACCUCA	8mer	-0.63	99	-0.63	5.414	0.95
hsa-let-7c-5p	TNFSF9	Position 92-99 of TNFSF9 3' UTR	5'-UCCCUGCUGCUUUCUCUACCUCA	8mer	-0.63	99	-0.63	5.414	0.95
hsa-let-7e-5p	TNFSF9	Position 92-99 of TNFSF9 3' UTR	5'-UCCCUGCUGCUUUCUCUACCUCA	8mer	-0.63	99	-0.63	5.414	0.95
hsa-let-7i-5p	TNFSF9	Position 92-99 of TNFSF9 3' UTR	5'-UCCCUGCUGCUUUCUCUACCUCA	8mer	-0.63	99	-0.63	5.414	0.95
hsa-miR-4500	TNFSF9	Position 92-99 of TNFSF9 3' UTR	5'-UCCCUGCUGCUUUCUCUACCUCA	8mer	-0.63	99	-0.63	5.414	0.95
hsa-miR-133a-3p.1	CSF2	Position 83-90 of CSF2 3' UTR	5'-GAUGGUCAUCUUGGAGGGACCAA	8mer	-0.57	99	-0.57	1.891	< 0.1
hsa-miR-760	IL6	Position 283-290 of IL6 3' UTR	5'-GAAUAUCCUUUGUUUCAGAGCCA	8mer	-0.57	99	-0.57	1.948	N/A
hsa-miR-149-5p	IL6	Position 285-292 of IL6 3' UTR	5'-AUAUCCUUUGUUUCAGAGCCAGA	8mer	-0.55	99	-0.55	1.808	N/A
hsa-miR-141-3p	STAT4	Position 237-244 of STAT4 3' UTR	5'-ACAACUUUAAGAAACCAGUGUUA	8mer	-0.51	99	-0.51	2.06	< 0.1
hsa-miR-200a-3p	STAT4	Position 237-244 of STAT4 3' UTR	5'-ACAACUUUAAGAAACCAGUGUUA	8mer	-0.5	99	-0.5	2.06	< 0.1
hsa-miR-135a-5p	STAT6	Position 1100-1107 of STAT6 3' UTR	5'-GGGGUCAUAGAUCCU--AAGCCAUA	8mer	-0.49	99	-0.04	5.351	0.92
hsa-miR-135b-5p	STAT6	Position 1100-1107 of STAT6 3' UTR	5'-GGGGUCAUAGAUCCU--AAGCCAUA	8mer	-0.49	99	-0.04	5.351	0.92
hsa-miR-138-5p	TNFSF4	Position 29-36 of TNFSF4 3' UTR	5'-AUAUCUAAAACCAGGCACCAGCA	8mer	-0.43	97	-0.43	2.7	0.34
hsa-miR-325-3p	TNFRSF18	Position 62-69 of TNFRSF18 3' UTR	5'-GGCUGCAGGGGACCU--UCAAUAAA	8mer	-0.4	98	-0.4	2.808	N/A
hsa-miR-326	TNFSF14	Position 555-562 of TNFSF14 3' UTR	5'-UACUAAGGACAAAAACCCAGAGA	8mer	-0.39	98	-0.39	2.182	N/A
hsa-miR-455-3p.1	CD80	Position 773-780 of CD80 3' UTR	5'-UUAACUGGUUCAUAUUGGACUGA	8mer	-0.39	98	-0.39	4.636	0.3
hsa-miR-130a-3p	TNF	Position 451-457 of TNF 3' UTR	5'-UCCCUCUAUUUAUGUUUGCACUU	7mer-m8	-0.38	98	-0.38	3.438	0.49
hsa-miR-326	CD8A	Position 361-368 of CD8A 3' UTR	5'-AGGGGGAGGCUAAAGCCCAGAGA	8mer	-0.38	98	-0.38	1.864	N/A
hsa-miR-141-3p	TGFB2	Position 109-116 of TGFB2 3' UTR	5'-AGAGCCUUGGUUCAUCAGUGUUA	8mer	-0.37	99	-0.37	5.541	0.81
hsa-miR-200a-3p	TGFB2	Position 109-116 of TGFB2 3' UTR	5'-AGAGCCUUGGUUCAUCAGUGUUA	8mer	-0.37	99	-0.37	5.541	0.81
hsa-miR-330-5p	TNFSF14	Position 555-562 of TNFSF14 3' UTR	5'-UACUAAGGACAAAAACCCAGAGA	8mer	-0.37	98	-0.37	2.182	N/A
hsa-miR-130b-3p	TNF	Position 451-457 of TNF 3' UTR	5'-UCCCUCUAUUUAUGU-UUGCACUU	7mer-m8	-0.36	98	-0.36	3.438	0.49
hsa-miR-454-3p	TNF	Position 451-457 of TNF 3' UTR	5'-UCCCUCUAUUUAUGUUUGCACUU	7mer-m8	-0.36	98	-0.36	3.438	0.49
hsa-miR-3666	TNF	Position 451-457 of TNF 3' UTR	5'-UCCCUCUAUUUAUGUUUGCACUU	7mer-m8	-0.35	98	-0.35	3.438	0.49
hsa-miR-6766-3p	CD226	Position 1993-2000 of CD226 3' UTR	5'-UUUCUGAAUGGUAAA-GACAAUCA	8mer	-0.35	96	0	1.924	< 0.1
hsa-let-7d-5p	IL10	Position 140-146 of IL10 3' UTR	5'-AUUUAUUACCUCUGAUACCUCAA	7mer-A1	-0.35	92	-0.35	6.938	0.95
hsa-miR-301a-3p	TNF	Position 451-457 of TNF 3' UTR	5'-UCCCUCUAUUUAUGUUUGCACUU	7mer-m8	-0.34	98	-0.34	3.438	0.49
hsa-miR-101-3p.1	IL13	Position 547-553 of IL13 3' UTR	5'-AGUCCCUGGUAAUAAGUACUGUG	7mer-m8	-0.34	97	-0.34	3.827	0.73
hsa-miR-135a-5p	GATA3	Position 207-213 of GATA3 3' UTR	5'-ACCCCAUCUGUGAAU---AAGCCAUU	7mer-m8	-0.34	97	-0.34	4.672	0.7
hsa-miR-135b-5p	GATA3	Position 207-213 of GATA3 3' UTR	5'-ACCCCAUCUGUGAAU---AAGCCAUU	7mer-m8	-0.34	97	-0.34	4.672	0.7
hsa-miR-301b-3p	TNF	Position 451-457 of TNF 3' UTR	5'-UCCCUCUAUUUAUGUUUGCACUU	7mer-m8	-0.34	97	-0.34	3.438	0.49
hsa-miR-4319	IFNG	Position 250-256 of IFNG 3' UTR	5'-AUUACAAGGCUUUAUCUCAGGGG	7mer-m8	-0.34	96	-0.34	3.121	0.3
hsa-miR-330-5p	CD8A	Position 361-368 of CD8A 3' UTR	5'-AGGGGGAGGCUAAAGCCCAGAGA	8mer	-0.33	97	-0.33	1.864	N/A
hsa-miR-219a-5p	CD226	Position 1993-2000 of CD226 3' UTR	5'-UUUCUGAAUGGUAAAGACAAUCA	8mer	-0.33	96	0	1.924	< 0.1
hsa-miR-4782-3p	CD226	Position 1993-2000 of CD226 3' UTR	5'-UUUCUGAAUGGUAAAGACAAUCA	8mer	-0.33	96	0	1.924	< 0.1
hsa-let-7e-5p	IL10	Position 140-146 of IL10 3' UTR	5'-AUUUAUUACCUCUGAUACCUCAA	7mer-A1	-0.33	91	-0.33	6.938	0.95
hsa-miR-4458	IL10	Position 140-146 of IL10 3' UTR	5'-AUUUAUUACCUCUGAUACCUCAA	7mer-A1	-0.33	91	-0.33	6.938	0.95
hsa-miR-15a-5p	CD163	Position 154-160 of CD163 3' UTR	5'-UAGGAUUCCUAAGACUGCUGCUG	7mer-m8	-0.32	96	-0.32	3.246	0.51
hsa-miR-15b-5p	CD163	Position 154-160 of CD163 3' UTR	5'-UAGGAUUCCUAAGACUGCUGCUG	7mer-m8	-0.32	96	-0.32	3.246	0.51
hsa-miR-16-5p	CD163	Position 154-160 of CD163 3' UTR	5'-UAGGAUUCCUAAGACUGCUGCUG	7mer-m8	-0.32	96	-0.32	3.246	0.51
hsa-miR-195-5p	CD163	Position 154-160 of CD163 3' UTR	5'-UAGGAUUCCUAAGACUGCUGCUG	7mer-m8	-0.32	96	-0.32	3.246	0.51
hsa-miR-125a-5p	VTCN1	Position 304-311 of VTCN1 3' UTR	5'-AGUGCAUCCCCAGAUCUCAGGGA	8mer	-0.32	96	-0.31	3.287	0.69
hsa-miR-125b-5p	VTCN1	Position 304-311 of VTCN1 3' UTR	5'-AGUGCAUCCCCAGAUCUCAGGGA	8mer	-0.32	96	-0.31	3.287	0.69
hsa-let-7a-5p	CD86	Position 435-441 of CD86 3' UTR	5'-CCUGUGACUAAACAA----CUACCUCC	7mer-m8	-0.32	91	-0.32	3.203	0.5
hsa-let-7b-5p	CD86	Position 435-441 of CD86 3' UTR	5'-CCUGUGACUAAACAA----CUACCUCC	7mer-m8	-0.32	91	-0.32	3.203	0.5
hsa-let-7b-5p	IL10	Position 140-146 of IL10 3' UTR	5'-AUUUAUUACCUCUGAUACCUCAA	7mer-A1	-0.32	91	-0.32	6.938	0.95
hsa-let-7c-5p	CD86	Position 435-441 of CD86 3' UTR	5'-CCUGUGACUAAACAA----CUACCUCC	7mer-m8	-0.32	91	-0.32	3.203	0.5
hsa-let-7e-5p	CD86	Position 435-441 of CD86 3' UTR	5'-CCUGUGACUAAACAA----CUACCUCC	7mer-m8	-0.32	91	-0.32	3.203	0.5
hsa-let-7f-5p	CD86	Position 435-441 of CD86 3' UTR	5'-CCUGUGACUAAACAA----CUACCUCC	7mer-m8	-0.32	91	-0.32	3.203	0.5
hsa-let-7g-5p	CD86	Position 435-441 of CD86 3' UTR	5'-CCUGUGACUAAACAACUACCUCC	7mer-m8	-0.32	91	-0.32	3.203	0.5
hsa-let-7i-5p	CD86	Position 435-441 of CD86 3' UTR	5'-CCUGUGACUAAACAACUACCUCC	7mer-m8	-0.32	91	-0.32	3.203	0.5
hsa-miR-4500	CD86	Position 435-441 of CD86 3' UTR	5'-CCUGUGACUAAACAACUACCUCC	7mer-m8	-0.32	91	-0.32	3.203	0.5
hsa-miR-98-5p	CD86	Position 435-441 of CD86 3' UTR	5'-CCUGUGACUAAACAA----CUACCUCC	7mer-m8	-0.32	91	-0.32	3.203	0.5
hsa-let-7a-5p	IL10	Position 140-146 of IL10 3' UTR	5'-AUUUAUUACCUCUGAUACCUCAA	7mer-A1	-0.32	90	-0.32	6.938	0.95
hsa-let-7c-5p	IL10	Position 140-146 of IL10 3' UTR	5'-AUUUAUUACCUCUGAUACCUCAA	7mer-A1	-0.32	90	-0.32	6.938	0.95
hsa-let-7f-5p	IL10	Position 140-146 of IL10 3' UTR	5'-AUUUAUUACCUCUGAUACCUCAA	7mer-A1	-0.32	90	-0.32	6.938	0.95
hsa-let-7g-5p	IL10	Position 140-146 of IL10 3' UTR	5'-AUUUAUUACCUCUGAUACCUCAA	7mer-A1	-0.32	90	-0.32	6.938	0.95
hsa-miR-98-5p	IL10	Position 140-146 of IL10 3' UTR	5'-AUUUAUUACCUCUGAUACCUCAA	7mer-A1	-0.32	90	-0.32	6.938	0.95
hsa-miR-4319	VTCN1	Position 304-311 of VTCN1 3' UTR	5'-AGUGCAUCCCCAGAUCUCAGGGA	8mer	-0.31	96	-0.31	3.287	0.69
hsa-miR-497-5p	CD163	Position 154-160 of CD163 3' UTR	5'-UAGGAUUCCUAAGACUGCUGCUG	7mer-m8	-0.31	96	-0.31	3.246	0.51
hsa-miR-6838-5p	CD163	Position 154-160 of CD163 3' UTR	5'-UAGGAUUCCUAAGACUGCUGCUG	7mer-m8	-0.31	96	-0.31	3.246	0.51
hsa-miR-125b-5p	IFNG	Position 250-256 of IFNG 3' UTR	5'-AUUACAAGGCUUUAUCUCAGGGG	7mer-m8	-0.31	95	-0.31	3.121	0.3
hsa-let-7i-5p	IL10	Position 140-146 of IL10 3' UTR	5'-AUUUAUUACCUCUGAUACCUCAA	7mer-A1	-0.31	89	-0.31	6.938	0.95
hsa-miR-136-5p	CD3G	Position 132-139 of CD3G 3' UTR	5'-UUCCCAGAGAUGACAAAUGGAGA	8mer	-0.3	97	-0.3	3.679	N/A
hsa-miR-4295	TNF	Position 451-457 of TNF 3' UTR	5'-UCCCUCUAUUUAUGUUUGCACUU	7mer-m8	-0.3	96	-0.3	3.438	0.49

(Continued)

miRNA	Target gene	Position of target region	Predicted target sequence	Site type	Context++ score	Context++ score percentile	Weighted context++ score	Conserved branch length	P_{CT}
hsa-let-7d-5p	CD86	Position 435-441 of CD86 3' UTR	5'-CCUGUGACUAAACAACUACCUCC	7mer-m8	-0.3	88	-0.3	3.203	0.5
hsa-miR-424-5p	CD163	Position 154-160 of CD163 3' UTR	5'-UAGGAUUCCUAAGACUGCUGCUG	7mer-m8	-0.29	95	-0.28	3.246	0.51
hsa-miR-5195-3p	CD40	Position 568-575 of CD40 3' UTR	5'-UUUGUGAUAGUGAACAACUGGAA	8mer	-0.29	94	-0.29	2.055	0.13
hsa-miR-29a-3p	TBX21	Position 245-251 of TBX21 3' UTR	5'-CUACAGUCGUUUACC-UGGUGCUG	7mer-m8	-0.29	92	-0.29	4.504	0.84
hsa-miR-29b-3p	TBX21	Position 245-251 of TBX21 3' UTR	5'-CUACAGUCGUUUACC-UGGUGCUG	7mer-m8	-0.29	92	-0.29	4.504	0.84
hsa-miR-29c-3p	TBX21	Position 245-251 of TBX21 3' UTR	5'-CUACAGUCGUUUACC-UGGUGCUG	7mer-m8	-0.29	92	-0.29	4.504	0.84
hsa-miR-4500	IL10	Position 140-146 of IL10 3' UTR	5'-AUUUAUUACCUCUGAUACCUCAA	7mer-A1	-0.29	88	-0.29	6.938	0.95
hsa-miR-372-3p	PDCD1LG2	Position 125-131 of PDCD1LG2 3' UTR	5'-GCCUUUGGAUGACCCAGCACUUU	7mer-m8	-0.28	97	-0.28	3.715	0.52
hsa-miR-125a-5p	IFNG	Position 250-256 of IFNG 3' UTR	5'-AUUACAAGGCUUUAUCUCAGGGG	7mer-m8	-0.28	95	-0.28	3.121	0.3
hsa-miR-5195-3p	CD40	Position 409-416 of CD40 3' UTR	5'-UUUGUGAUAGUGAACAACUGGAA	8mer	-0.28	93	-0.28	2.055	0.13
hsa-miR-133a-3p.1	TNFRSF14	Position 180-186 of TNFRSF14 3' UTR	5'-ACGAAGGCCGGAGCUGGGACCAG	7mer-m8	-0.28	89	-0.28	3.253	N/A
hsa-miR-4458	CD86	Position 435-441 of CD86 3' UTR	5'-CCUGUGACUAAACAACUACCUCC	7mer-m8	-0.28	85	-0.28	3.203	0.5
hsa-miR-302a-3p	PDCD1LG2	Position 125-131 of PDCD1LG2 3' UTR	5'-GCCUUUGGAUGACCCAGCACUUU	7mer-m8	-0.27	96	-0.27	3.715	0.52
hsa-miR-302b-3p	PDCD1LG2	Position 125-131 of PDCD1LG2 3' UTR	5'-GCCUUUGGAUGACCCAGCACUUU	7mer-m8	-0.27	96	-0.27	3.715	0.52
hsa-miR-302c-3p.1	PDCD1LG2	Position 125-131 of PDCD1LG2 3' UTR	5'-GCCUUUGGAUGACCCAGCACUUU	7mer-m8	-0.27	96	-0.27	3.715	0.52
hsa-miR-302d-3p	PDCD1LG2	Position 125-131 of PDCD1LG2 3' UTR	5'-GCCUUUGGAUGACCCAGCACUUU	7mer-m8	-0.27	96	-0.27	3.715	0.52
hsa-miR-302e	PDCD1LG2	Position 125-131 of PDCD1LG2 3' UTR	5'-GCCUUUGGAUGACCCAGCACUUU	7mer-m8	-0.27	96	-0.27	3.715	0.52
hsa-miR-15a-5p	CD163	Position 132-138 of CD163 3' UTR	5'-UAGGAUUCCUAAGACUGCUGCUG	7mer-m8	-0.27	93	-0.26	3.246	0.51
hsa-miR-15b-5p	CD163	Position 132-138 of CD163 3' UTR	5'-UAGGAUUCCUAAGACUGCUGCUG	7mer-m8	-0.27	93	-0.26	3.246	0.51
hsa-miR-16-5p	CD163	Position 132-138 of CD163 3' UTR	5'-UAGGAUUCCUAAGACUGCUGCUG	7mer-m8	-0.27	93	-0.26	3.246	0.51
hsa-miR-195-5p	CD163	Position 132-138 of CD163 3' UTR	5'-UAGGAUUCCUAAGACUGCUGCUG	7mer-m8	-0.27	93	-0.26	3.246	0.51
hsa-miR-325-3p	TNFRSF18	Position 315-322 of TNFRSF18 3' UTR	5'-GGCUGCAGGGGACCU--UCAAUAAA	8mer	-0.26	96	-0.26	2.808	N/A
hsa-miR-6838-5p	CD163	Position 132-138 of CD163 3' UTR	5'-UAGGAUUCCUAAGACUGCUGCUG	7mer-m8	-0.26	92	-0.25	3.246	0.51
hsa-miR-520b	PDCD1LG2	Position 125-131 of PDCD1LG2 3' UTR	5'-GCCUUUGGAUGACCCAGCACUUU	7mer-m8	-0.25	95	-0.25	3.715	0.52
hsa-miR-520c-3p	PDCD1LG2	Position 125-131 of PDCD1LG2 3' UTR	5'-GCCUUUGGAUGACCCAGCACUUU	7mer-m8	-0.25	95	-0.25	3.715	0.52
hsa-miR-520e	PDCD1LG2	Position 125-131 of PDCD1LG2 3' UTR	5'-GCCUUUGGAUGACCCAGCACUUU	7mer-m8	-0.25	95	-0.25	3.715	0.52
hsa-miR-497-5p	CD163	Position 132-138 of CD163 3' UTR	5'-UAGGAUUCCUAAGACUGCUGCUG	7mer-m8	-0.25	93	-0.25	3.246	0.51
hsa-miR-296-3p	CD40LG	Position 97-103 of CD40LG 3' UTR	5'-UAACUGCCUAUUUAUAACCCUAG	7mer-A1	-0.24	95	-0.24	3.812	N/A
hsa-miR-520d-3p	PDCD1LG2	Position 125-131 of PDCD1LG2 3' UTR	5'-GCCUUUGGAUGACCCAGCACUUU	7mer-m8	-0.24	95	-0.24	3.715	0.52
hsa-miR-532-5p	CD40LG	Position 141-147 of CD40LG 3' UTR	5'-UUUAUUAUACACUCCAAGGCAUG	7mer-m8	-0.24	95	-0.24	2.835	N/A
hsa-miR-143-3p	HHLA2	Position 56-62 of HHLA2 3' UTR	5'-UAAUAUCCAGUGACUUCAUCUCC	7mer-m8	-0.23	95	-0.23	3.151	0.24
hsa-miR-145-5p	CD40	Position 568-575 of CD40 3' UTR	5'-UUUGUGAUAGUGAACAACUGGAA	8mer	-0.23	94	-0.23	2.055	0.13
hsa-miR-424-5p	CD163	Position 132-138 of CD163 3' UTR	5'-UAGGAUUCCUAAGACUGCUGCUG	7mer-m8	-0.23	91	-0.23	3.246	0.51
hsa-miR-520a-3p	PDCD1LG2	Position 125-131 of PDCD1LG2 3' UTR	5'-GCCUUUGGAUGACCCAGCACUUU	7mer-m8	-0.22	94	-0.22	3.715	0.52
hsa-miR-145-5p	CD40	Position 409-416 of CD40 3' UTR	5'-UUUGUGAUAGUGAACAACUGGAA	8mer	-0.22	93	-0.22	2.055	0.13
hsa-miR-155-5p	CTLA4	Position 296-302 of CTLA4 3' UTR	5'-UUAAUAUGGGGAUGCAGCAUUAU	7mer-m8	-0.22	90	-0.22	3.27	0.23
hsa-miR-15a-5p	IL2RB	Position 182-188 of IL2RB 3' UTR	5'-UUUCCAAACCUCCAC-UGCUGCUC	7mer-m8	-0.22	89	-0.22	4.494	0.67
hsa-miR-182-5p	CD96	Position 392-399 of CD96 3' UTR	5'-AACAUUGUAAACUCUUUGCCAAA	8mer	-0.22	88	-0.22	2.463	0.21
hsa-miR-140-3p.1	IL12RB2	Position 448-454 of IL12RB2 3' UTR	5'-ACCCACUCACCUUCU--CCUGUGGU	7mer-m8	-0.21	93	-0.21	3.082	N/A
hsa-miR-140-3p.2	CD274	Position 43-49 of CD274 3' UTR	5'-GCAGGGAUUCUCAACCUGUGGUU	7mer-m8	-0.21	93	-0.21	3.082	0.21
hsa-miR-15b-5p	IL2RB	Position 182-188 of IL2RB 3' UTR	5'-UUUCCAAACCUCCAC-UGCUGCUC	7mer-m8	-0.21	88	-0.21	4.494	0.67
hsa-miR-26a-5p	ICOS	Position 58-64 of ICOS 3' UTR	5'-GGCCAGUUUUCCUCA---ACUUGAAG	7mer-A1	-0.2	93	-0.2	3.645	0.41
hsa-miR-26b-5p	ICOS	Position 58-64 of ICOS 3' UTR	5'-GGCCAGUUUUCCUCA---ACUUGAAG	7mer-A1	-0.2	93	-0.2	3.645	0.41
hsa-miR-181b-5p	CD4	Position 74-81 of CD4 3' UTR	5'-UGCCUGCGGACCAGA-UGAAUGUA	8mer	-0.2	92	-0.2	4.488	0.69
hsa-miR-181c-5p	CD4	Position 74-81 of CD4 3' UTR	5'-UGCCUGCGGACCAGAUGAAUGUA	8mer	-0.2	92	-0.2	4.488	0.69
hsa-miR-4770	HHLA2	Position 56-62 of HHLA2 3' UTR	5'-UAAUAUCCAGUGACUUCAUCUCC	7mer-m8	-0.2	92	-0.2	3.151	0.24
hsa-miR-181a-5p	CD4	Position 74-81 of CD4 3' UTR	5'-UGCCUGCGGACCAGA-UGAAUGUA	8mer	-0.2	91	-0.2	4.488	0.69
hsa-miR-181d-5p	CD4	Position 74-81 of CD4 3' UTR	5'-UGCCUGCGGACCAGA-UGAAUGUA	8mer	-0.2	91	-0.2	4.488	0.69
hsa-miR-497-5p	IL2RB	Position 182-188 of IL2RB 3' UTR	5'-UUUCCAAACCUCCAC-UGCUGCUC	7mer-m8	-0.2	88	-0.2	4.494	0.67
hsa-miR-373-3p	PDCD1LG2	Position 125-131 of PDCD1LG2 3' UTR	5'-GCCUUUGGAUGACCCAGCACUUU	7mer-m8	-0.19	96	-0.19	3.715	0.52
hsa-miR-26a-5p	ICOS	Position 50-56 of ICOS 3' UTR	5'-GGCCAGUUUUCCUCA---ACUUGAAG	7mer-A1	-0.19	93	-0.19	3.645	0.41
hsa-miR-26b-5p	ICOS	Position 50-56 of ICOS 3' UTR	5'-GGCCAGUUUUCCUCA---ACUUGAAG	7mer-A1	-0.19	93	-0.19	3.645	0.41
hsa-miR-425-5p	TGFB1	Position 151-157 of TGFB1 3' UTR	5'-GGACUGCGGAUCUCUGUGUCAUU	7mer-m8	-0.19	93	0	3.093	< 0.1
hsa-miR-15a-5p	PDCD1	Position 305-311 of PDCD1 3' UTR	5'-AGCUCCCUGAAUCUCUGCUGCUG	7mer-m8	-0.19	86	-0.19	2.877	0.44
hsa-miR-15b-5p	PDCD1	Position 305-311 of PDCD1 3' UTR	5'-AGCUCCCUGAAUCUCUGCUGCUG	7mer-m8	-0.19	86	-0.19	2.877	0.44
hsa-miR-16-5p	PDCD1	Position 305-311 of PDCD1 3' UTR	5'-AGCUCCCUGAAUCUCUGCUGCUG	7mer-m8	-0.19	86	-0.19	2.877	0.44
hsa-miR-195-5p	PDCD1	Position 305-311 of PDCD1 3' UTR	5'-AGCUCCCUGAAUCUCUGCUGCUG	7mer-m8	-0.19	86	-0.19	2.877	0.44
hsa-miR-424-5p	PDCD1	Position 305-311 of PDCD1 3' UTR	5'-AGCUCCCUGAAUCUCUGCUGCUG	7mer-m8	-0.19	86	-0.19	2.877	0.44
hsa-miR-142-3p.1	BTLA	Position 370-376 of BTLA 3' UTR	5'-UUACCUGCAUCAGUUCACUACAC	7mer-A1	-0.19	82	-0.19	5.172	0.68
hsa-miR-4465	ICOS	Position 58-64 of ICOS 3' UTR	5'-GGCCAGUUUUCCUCAACUUGAAG	7mer-A1	-0.18	91	-0.18	3.645	0.41
hsa-miR-4465	ICOS	Position 50-56 of ICOS 3' UTR	5'-GGCCAGUUUUCCUCAACUUGAAG	7mer-A1	-0.18	91	-0.18	3.645	0.41
hsa-miR-6088	HHLA2	Position 56-62 of HHLA2 3' UTR	5'-UAAUAUCCAGUGACUUCAUCUCC	7mer-m8	-0.18	91	-0.18	3.151	0.24
hsa-miR-497-5p	PDCD1	Position 305-311 of PDCD1 3' UTR	5'-AGCUCCCUGAAUCUCUGCUGCUG	7mer-m8	-0.18	86	-0.18	2.877	0.44
hsa-miR-424-5p	IL2RB	Position 182-188 of IL2RB 3' UTR	5'-UUUCCAAACCUCCACUGCUGCUC	7mer-m8	-0.18	85	-0.18	4.494	0.67
hsa-miR-6838-5p	PDCD1	Position 305-311 of PDCD1 3' UTR	5'-AGCUCCCUGAAUCUCUGCUGCUG	7mer-m8	-0.18	85	-0.18	2.877	0.44
hsa-miR-1297	ICOS	Position 58-64 of ICOS 3' UTR	5'-GGCCAGUUUUCCUCAACUUGAAG	7mer-A1	-0.17	91	-0.17	3.645	0.41
hsa-miR-1297	ICOS	Position 50-56 of ICOS 3' UTR	5'-GGCCAGUUUUCCUCAACUUGAAG	7mer-A1	-0.17	91	-0.17	3.645	0.41
hsa-miR-16-5p	IL2RB	Position 182-188 of IL2RB 3' UTR	5'-UUUCCAAACCUCCACUGCUGCUC	7mer-m8	-0.17	84	-0.17	4.494	0.67
hsa-miR-195-5p	IL2RB	Position 182-188 of IL2RB 3' UTR	5'-UUUCCAAACCUCCACUGCUGCUC	7mer-m8	-0.17	84	-0.17	4.494	0.67
hsa-miR-340-5p	IL4	Position 52-58 of IL4 3' UTR	5'-UUAAGUAUUUAUAUAUUUAUAAC	7mer-A1	-0.16	98	-0.16	4.169	N/A
hsa-miR-6835-3p	TNFSF18	Position 615-622 of TNFSF18 3' UTR	5'-UCCUCAGGAUGCAGGGUGCUUUA	8mer	-0.16	95	-0.16	1.975	N/A
hsa-miR-15a-5p	PDCD1	Position 308-314 of PDCD1 3' UTR	5'-UCCCUGAAUCUCUGCUGCUGCUG	7mer-m8	-0.16	82	-0.16	2.877	0.44
hsa-miR-15b-5p	PDCD1	Position 308-314 of PDCD1 3' UTR	5'-UCCCUGAAUCUCUGCUGCUGCUG	7mer-m8	-0.16	82	-0.16	2.877	0.44
hsa-miR-16-5p	PDCD1	Position 308-314 of PDCD1 3' UTR	5'-UCCCUGAAUCUCUGCUGCUGCUG	7mer-m8	-0.16	82	-0.16	2.877	0.44

S3 Table

Takashima et al.

(Continued)

miRNA	Target gene	Position of target region	Predicted target sequence	Site type	Context++ score	Context++ score percentile	Weighted context++ score	Conserved branch length	P_{CT}
hsa-miR-195-5p	PDCD1	Position 308-314 of PDCD1 3' UTR	5'-UCCCUGAAUCUCUGCUGCUGCUG	7mer-m8	-0.16	82	-0.16	2.877	0.44
hsa-miR-6838-5p	IL2RB	Position 182-188 of IL2RB 3' UTR	5'-UUUCCAAACCUCCACUGCUGCUC	7mer-m8	-0.16	82	-0.16	4.494	0.67
hsa-miR-6838-5p	PDCD1	Position 308-314 of PDCD1 3' UTR	5'-UCCCUGAAUCUCUGCUGCUGCUG	7mer-m8	-0.16	81	-0.16	2.877	0.44
hsa-miR-141-3p	TGFB2	Position 300-306 of TGFB2 3' UTR	5'-CUGGAAGAAUUUAUUAGUGUUAA	7mer-A1	-0.15	88	-0.15	5.221	0.46
hsa-miR-7153-5p	CD3D	Position 2339-2346 of CD3D 3' UTR	5'-CAUCCCUCAUUCCCCAGUUCUCA	8mer	-0.15	86	0	1.878	< 0.1
hsa-miR-424-5p	PDCD1	Position 308-314 of PDCD1 3' UTR	5'-UCCCUGAAUCUCUGCUGCUGCUG	7mer-m8	-0.15	80	-0.15	2.877	0.44
hsa-miR-378a-3p	CD3G	Position 17-23 of CD3G 3' UTR	5'-CUCAGGACUCAGAGUAGUCCAGG	7mer-m8	-0.14	79	-0.14	5.231	N/A
hsa-miR-378c	CD3G	Position 17-23 of CD3G 3' UTR	5'-CUCAGGACUCAGAGUAGUCCAGG	7mer-m8	-0.14	79	-0.14	5.231	N/A
hsa-miR-378d	CD3G	Position 17-23 of CD3G 3' UTR	5'-CUCAGGACUCAGAGUAGUCCAGG	7mer-m8	-0.14	79	-0.14	5.231	N/A
hsa-miR-378e	CD3G	Position 17-23 of CD3G 3' UTR	5'-CUCAGGACUCAGAGUAGUCCAGG	7mer-m8	-0.14	79	-0.14	5.231	N/A
hsa-miR-378i	CD3G	Position 17-23 of CD3G 3' UTR	5'-CUCAGGACUCAGAGUAGUCCAGG	7mer-m8	-0.14	79	-0.14	5.231	N/A
hsa-miR-410-3p	IL4	Position 45-51 of IL4 3' UTR	5'-UUAUUUUUUAAGUAUUUAUAUAU	7mer-A1	-0.13	97	-0.13	4.241	N/A
hsa-miR-200a-3p	TGFB2	Position 300-306 of TGFB2 3' UTR	5'-CUGGAAGAAUUUAUUAGUGUUAA	7mer-A1	-0.13	87	-0.13	5.221	0.46
hsa-miR-16-5p	PDCD1	Position 311-317 of PDCD1 3' UTR	5'-CUGAAUCUCUGCUGCUGCUGCUG	7mer-m8	-0.13	78	-0.13	2.877	0.44
hsa-miR-195-5p	PDCD1	Position 311-317 of PDCD1 3' UTR	5'-CUGAAUCUCUGCUGCUGCUGCUG	7mer-m8	-0.13	78	-0.13	2.877	0.44
hsa-miR-155-5p	CTLA4	Position 259-265 of CTLA4 3' UTR	5'-UUAAUAUGGGGAUGCAGCAUUAU	7mer-m8	-0.13	77	-0.13	3.27	0.23
hsa-miR-6838-5p	PDCD1	Position 311-317 of PDCD1 3' UTR	5'-CUGAAUCUCUGCUGCUGCUGCUG	7mer-m8	-0.13	77	-0.13	2.877	0.44
hsa-miR-146a-5p	CD3D	Position 2339-2346 of CD3D 3' UTR	5'-CAUCCCUCAUUCCCCAGUUCUCA	8mer	-0.12	81	0	1.878	< 0.1
hsa-miR-497-5p	PDCD1	Position 308-314 of PDCD1 3' UTR	5'-UCCCUGAAUCUCUGCUGCUGCUG	7mer-m8	-0.12	77	-0.12	2.877	0.44
hsa-miR-15b-5p	PDCD1	Position 311-317 of PDCD1 3' UTR	5'-CUGAAUCUCUGCUGCUGCUGCUG	7mer-m8	-0.12	75	-0.12	2.877	0.44
hsa-miR-146b-5p	CD3D	Position 2339-2346 of CD3D 3' UTR	5'-CAUCCCUCAUUCCCCAGUUCUCA	8mer	-0.11	80	0	1.878	< 0.1
hsa-miR-378h	CD3G	Position 17-23 of CD3G 3' UTR	5'-CUCAGGACUCAGAGUAGUCCAGG	7mer-m8	-0.11	75	-0.11	5.231	N/A
hsa-miR-422a	CD3G	Position 17-23 of CD3G 3' UTR	5'-CUCAGGACUCAGAGUAGUCCAGG	7mer-m8	-0.11	75	-0.11	5.231	N/A
hsa-miR-424-5p	PDCD1	Position 311-317 of PDCD1 3' UTR	5'-CUGAAUCUCUGCUGCUGCUGCUG	7mer-m8	-0.11	74	-0.11	2.877	0.44
hsa-miR-15a-5p	PDCD1	Position 311-317 of PDCD1 3' UTR	5'-CUGAAUCUCUGCUGCUGCUGCUG	7mer-m8	-0.11	73	-0.11	2.877	0.44
hsa-miR-4644	CD4	Position 300-306 of CD4 3' UTR	5'-UCUCCUCACCCAGGGUCUCUCCC	7mer-m8	-0.11	73	-0.11	3.09	N/A
hsa-miR-4262	CD4	Position 74-81 of CD4 3' UTR	5'-UGCCUGCGGACCAGAUGAAUGUA	8mer	-0.1	88	-0.1	4.488	0.69
hsa-miR-325-3p	TNFRSF4	Position 192-198 of TNFRSF4 3' UTR	5'-UGCCCCGCGGGACCACAAUAAAA	7mer-A1	-0.1	82	-0.1	4.787	N/A
hsa-miR-497-5p	PDCD1	Position 311-317 of PDCD1 3' UTR	5'-CUGAAUCUCUGCUGCUGCUGCUG	7mer-m8	-0.1	72	-0.1	2.877	0.44
hsa-miR-6838-5p	PDCD1	Position 314-320 of PDCD1 3' UTR	5'-AAUCUCUGCUGCUGCUGCUGCUG	7mer-m8	-0.1	72	-0.1	2.877	0.44
hsa-miR-6838-5p	PDCD1	Position 317-323 of PDCD1 3' UTR	5'-CUCUGCUGCUGCUGCUGCUGCUG	7mer-m8	-0.1	72	-0.1	2.877	0.44
hsa-miR-4306	CD4	Position 300-306 of CD4 3' UTR	5'-UCUCCUCACCCAGGGUCUCUCCC	7mer-m8	-0.1	71	-0.1	3.09	N/A
hsa-miR-185-5p	CD4	Position 300-306 of CD4 3' UTR	5'-UCUCCUCACCCAGGGUCUCUCCC	7mer-m8	-0.1	70	-0.1	3.09	N/A
hsa-miR-124-3p.1	CD276	Position 262-268 of CD276 3' UTR	5'-AGUCAUCCUGCCUGCUGCCUUAU	7mer-A1	-0.09	80	-0.08	3.893	0.7
hsa-miR-195-5p	PDCD1	Position 314-320 of PDCD1 3' UTR	5'-AAUCUCUGCUGCUGCUGCUGCUG	7mer-m8	-0.09	71	-0.09	2.877	0.44
hsa-miR-195-5p	PDCD1	Position 317-323 of PDCD1 3' UTR	5'-CUCUGCUGCUGCUGCUGCUGCUG	7mer-m8	-0.09	70	-0.09	2.877	0.44
hsa-miR-378b	CD3G	Position 17-23 of CD3G 3' UTR	5'-CUCAGGACUCAGAGUAGUCCAGG	7mer-m8	-0.09	70	-0.09	5.231	N/A
hsa-miR-378f	CD3G	Position 17-23 of CD3G 3' UTR	5'-CUCAGGACUCAGAGUAGUCCAGG	7mer-m8	-0.09	70	-0.09	5.231	N/A
hsa-miR-15b-5p	PDCD1	Position 314-320 of PDCD1 3' UTR	5'-AAUCUCUGCUGCUGCUGCUGCUG	7mer-m8	-0.09	69	-0.09	2.877	0.44
hsa-miR-15b-5p	PDCD1	Position 317-323 of PDCD1 3' UTR	5'-CUCUGCUGCUGCUGCUGCUGCUG	7mer-m8	-0.09	69	-0.09	2.877	0.44
hsa-miR-6838-5p	PDCD1	Position 320-326 of PDCD1 3' UTR	5'-UGCUGCUGCUGCUGCUGCUGCUG	7mer-m8	-0.09	68	-0.09	2.877	0.44
hsa-miR-6838-5p	PDCD1	Position 323-329 of PDCD1 3' UTR	5'-UGCUGCUGCUGCUGCUGCUGCUG	7mer-m8	-0.09	68	-0.09	2.877	0.44
hsa-miR-203a-3p.1	TGFB3	Position 155-161 of TGFB3 3' UTR	5'-AGGUUUCCUUUUGGAACAUUUCU	7mer-m8	-0.08	82	-0.08	4.012	0.23
hsa-miR-15a-5p	PDCD1	Position 314-320 of PDCD1 3' UTR	5'-AAUCUCUGCUGCUGCUGCUGCUG	7mer-m8	-0.08	67	-0.08	2.877	0.44
hsa-miR-16-5p	PDCD1	Position 317-323 of PDCD1 3' UTR	5'-CUCUGCUGCUGCUGCUGCUGCUG	7mer-m8	-0.08	67	-0.08	2.877	0.44
hsa-miR-195-5p	PDCD1	Position 323-329 of PDCD1 3' UTR	5'-UGCUGCUGCUGCUGCUGCUGCUG	7mer-m8	-0.08	67	-0.08	2.877	0.44
hsa-miR-424-5p	PDCD1	Position 314-320 of PDCD1 3' UTR	5'-AAUCUCUGCUGCUGCUGCUGCUG	7mer-m8	-0.08	67	-0.08	2.877	0.44
hsa-miR-424-5p	PDCD1	Position 317-323 of PDCD1 3' UTR	5'-CUCUGCUGCUGCUGCUGCUGCUG	7mer-m8	-0.08	67	-0.08	2.877	0.44
hsa-miR-15a-5p	PDCD1	Position 317-323 of PDCD1 3' UTR	5'-CUCUGCUGCUGCUGCUGCUGCUG	7mer-m8	-0.08	66	-0.08	2.877	0.44
hsa-miR-15b-5p	PDCD1	Position 323-329 of PDCD1 3' UTR	5'-UGCUGCUGCUGCUGCUGCUGCUG	7mer-m8	-0.08	65	-0.08	2.877	0.44
hsa-miR-653-5p	CD3G	Position 203-209 of CD3G 3' UTR	5'-UAAAAACAAAUACUGUGUUUCAG	7mer-m8	-0.07	76	-0.07	3.983	N/A
hsa-miR-23a-3p	TIGIT	Position 1309-1316 of TIGIT 3' UTR	5'-AAGGAACUGAAAGAGAAUGUGAA	8mer	-0.07	72	-0.07	2.612	< 0.1
hsa-miR-23c	TIGIT	Position 1309-1316 of TIGIT 3' UTR	5'-AAGGAACUGAAAGAGAAUGUGAA	8mer	-0.07	71	-0.07	2.612	< 0.1
hsa-miR-195-5p	PDCD1	Position 320-326 of PDCD1 3' UTR	5'-UGCUGCUGCUGCUGCUGCUGCUG	7mer-m8	-0.07	66	-0.07	2.877	0.44
hsa-miR-15b-5p	PDCD1	Position 320-326 of PDCD1 3' UTR	5'-UGCUGCUGCUGCUGCUGCUGCUG	7mer-m8	-0.07	64	-0.07	2.877	0.44
hsa-miR-16-5p	PDCD1	Position 314-320 of PDCD1 3' UTR	5'-AAUCUCUGCUGCUGCUGCUGCUG	7mer-m8	-0.07	64	-0.07	2.877	0.44
hsa-miR-497-5p	PDCD1	Position 314-320 of PDCD1 3' UTR	5'-AAUCUCUGCUGCUGCUGCUGCUG	7mer-m8	-0.07	64	-0.07	2.877	0.44
hsa-miR-497-5p	PDCD1	Position 317-323 of PDCD1 3' UTR	5'-CUCUGCUGCUGCUGCUGCUGCUG	7mer-m8	-0.07	64	-0.07	2.877	0.44
hsa-miR-16-5p	PDCD1	Position 323-329 of PDCD1 3' UTR	5'-UGCUGCUGCUGCUGCUGCUGCUG	7mer-m8	-0.07	63	-0.07	2.877	0.44
hsa-miR-15a-5p	PDCD1	Position 323-329 of PDCD1 3' UTR	5'-UGCUGCUGCUGCUGCUGCUGCUG	7mer-m8	-0.07	62	-0.07	2.877	0.44
hsa-miR-325-3p	LTA	Position 609-615 of LTA 3' UTR	5'-ACCCUCGAUGAAGCCCAAUAAAC	7mer-A1	-0.06	74	-0.06	4.819	N/A
hsa-miR-23b-3p	TIGIT	Position 1309-1316 of TIGIT 3' UTR	5'-AAGGAACUGAAAGAGAAUGUGAA	8mer	-0.06	68	-0.06	2.612	< 0.1
hsa-miR-16-5p	PDCD1	Position 320-326 of PDCD1 3' UTR	5'-UGCUGCUGCUGCUGCUGCUGCUG	7mer-m8	-0.06	62	-0.06	2.877	0.44
hsa-miR-424-5p	PDCD1	Position 323-329 of PDCD1 3' UTR	5'-UGCUGCUGCUGCUGCUGCUGCUG	7mer-m8	-0.06	62	-0.06	2.877	0.44
hsa-miR-424-5p	PDCD1	Position 320-326 of PDCD1 3' UTR	5'-UGCUGCUGCUGCUGCUGCUGCUG	7mer-m8	-0.06	61	-0.06	2.877	0.44
hsa-miR-15a-5p	PDCD1	Position 320-326 of PDCD1 3' UTR	5'-UGCUGCUGCUGCUGCUGCUGCUG	7mer-m8	-0.06	60	-0.06	2.877	0.44
hsa-miR-186-5p	IL2	Position 145-151 of IL2 3' UTR	5'-UAUGGAUCUUUUAUGAUUCUUUU	7mer-m8	-0.05	93	-0.05	3.057	N/A
hsa-miR-374a-5p	CD40LG	Position 128-134 of CD40LG 3' UTR	5'-UUAUGGAGAACUAUUUAUUAUAC	7mer-m8	-0.05	82	-0.05	2.856	N/A
hsa-miR-130a-5p	TIGIT	Position 1309-1316 of TIGIT 3' UTR	5'-AAGGAACUGAAAGAGAAUGUGAA	8mer	-0.05	65	-0.05	2.612	< 0.1
hsa-miR-497-5p	PDCD1	Position 323-329 of PDCD1 3' UTR	5'-UGCUGCUGCUGCUGCUGCUGCUG	7mer-m8	-0.05	58	-0.05	2.877	0.44
hsa-miR-497-5p	PDCD1	Position 320-326 of PDCD1 3' UTR	5'-UGCUGCUGCUGCUGCUGCUGCUG	7mer-m8	-0.05	56	-0.05	2.877	0.44
hsa-miR-374b-5p	CD40LG	Position 128-134 of CD40LG 3' UTR	5'-UUAUGGAGAACUAUUUAUUAUAC	7mer-m8	-0.04	75	-0.04	2.856	N/A
hsa-miR-485-5p	IL18R1	Position 536-543 of IL18R1 3' UTR	5'-GAGGCUGCAGGGCCUCAGCCUCA	8mer	-0.03	33	-0.03	1.905	N/A
hsa-miR-6884-5p	IL18R1	Position 536-543 of IL18R1 3' UTR	5'-GAGGCUGCAGGGCCUCAGCCUCA	8mer	-0.03	32	-0.03	1.905	N/A

S3 Table

Takashima et al.

(Continued)

miRNA	Target gene	Position of target region	Predicted target sequence	Site type	Context++ score	Context++ score percentile	Weighted context++ score	Conserved branch length	P_{CT}
hsa-miR-30a-5p	CEACAM1	Position 1455-1462 of CEACAM1 3' UTR	5'-UCAUGCUGGUGGCAUUGUUUACA	8mer	-0.03	28	-0.03	2.701	0.22
hsa-miR-30a-5p	CEACAM1	Position 1322-1329 of CEACAM1 3' UTR	5'-UCAUGCUGGUGGCAUUGUUUACA	8mer	-0.03	28	-0.03	2.701	0.22
hsa-miR-30d-5p	CEACAM1	Position 1455-1462 of CEACAM1 3' UTR	5'-UCAUGCUGGUGGCAUUGUUUACA	8mer	-0.03	28	-0.03	2.701	0.22
hsa-miR-30d-5p	CEACAM1	Position 1322-1329 of CEACAM1 3' UTR	5'-UCAUGCUGGUGGCAUUGUUUACA	8mer	-0.03	28	-0.03	2.701	0.22
hsa-miR-30b-5p	CEACAM1	Position 1455-1462 of CEACAM1 3' UTR	5'-UCAUGCUGGUGGCAU-UGUUUACA	8mer	-0.03	27	-0.03	2.701	0.22
hsa-miR-30b-5p	CEACAM1	Position 1322-1329 of CEACAM1 3' UTR	5'-UCAUGCUGGUGGCAU-UGUUUACA	8mer	-0.03	27	-0.03	2.701	0.22
hsa-miR-30c-5p	CEACAM1	Position 1455-1462 of CEACAM1 3' UTR	5'-UCAUGCUGGUGGCAU-UGUUUACA	8mer	-0.03	27	-0.03	2.701	0.22
hsa-miR-30c-5p	CEACAM1	Position 1322-1329 of CEACAM1 3' UTR	5'-UCAUGCUGGUGGCAU-UGUUUACA	8mer	-0.03	27	-0.03	2.701	0.22
hsa-miR-30e-5p	CEACAM1	Position 1455-1462 of CEACAM1 3' UTR	5'-UCAUGCUGGUGGCAUUGUUUACA	8mer	-0.03	26	-0.03	2.701	0.22
hsa-miR-30e-5p	CEACAM1	Position 1322-1329 of CEACAM1 3' UTR	5'-UCAUGCUGGUGGCAUUGUUUACA	8mer	-0.03	26	-0.03	2.701	0.22
hsa-miR-494-3p	TNFRSF9	Position 4795-4801 of TNFRSF9 3' UTR	5'-UUCCCCCACAGGAAGAUGUUUCC	7mer-m8	-0.02	51	0	2.998	N/A
hsa-miR-494-3p	CD3G	Position 203-209 of CD3G 3' UTR	5'-UAAAAACAAAUACUGUGUUUCAG	7mer-A1	-0.02	33	-0.01	3.983	N/A
hsa-miR-340-5p	CD40LG	Position 92-98 of CD40LG 3' UTR	5'-CCUGUUAACUGCCUAUUUAUAAC	7mer-A1	-0.01	51	-0.01	4.897	N/A
hsa-miR-129-5p	STAT1	Position 210-216 of STAT1 3' UTR	5'-AACAUGACCCUAUCA-CAAAAAAC	7mer-A1	-0.01	47	-0.01	3.602	N/A
hsa-miR-325-3p	FOXP3	Position 872-878 of FOXP3 3' UTR	5'-CCACAGUACCGUCCCCAAUAAAC	7mer-A1	-0.01	24	-0.01	4.15	N/A
hsa-miR-202-5p	CD28	Position 65-71 of CD28 3' UTR	5'-AUAUCACUGCUCUGGAUAGGAAA	7mer-A1	-0.01	23	-0.01	5.672	< 0.1

Note: TargetScanHuman 7.2 (http://www.targetscan.org/vert_72/).

S4 Table. Cox hazard regression analysis for the cancer immunity-related miRNAs in PCNSL.

	coef	se(coef)	z	p-value	HR (univariable)	HR (multivariable)
(Cancer immunity-related miRNAs)						
hsa-miR-1202	0.53	0.158	3.350554	0.001	1.13 (1.05-1.21, p=0.001)	1.70 (1.25-2.32, p=0.001)
hsa-miR-101	-7.628	2.62	-2.911913	0.004	0.18 (0.05-0.70, p=0.014)	0.00 (0.00-0.08, p=0.004)
hsa-miR-372	3.333	1.165	2.861809	0.004	0.97 (0.50-1.89, p=0.936)	28.02 (2.86-274.61, p=0.004)
hsa-miR-609	-3.616	1.49	-2.426783	0.015	0.64 (0.19-2.24, p=0.489)	0.03 (0.00-0.50, p=0.015)
(Th-1)						
hsa-miR-577	6.893	1.848	3.73044	<0.001	6.06 (1.32-27.86, p=0.021)	985.74 (26.35-36870.37, p<0.001)
hsa-miR-454	-4.513	1.373	-3.287874	0.001	0.74 (0.25-2.22, p=0.597)	0.01 (0.00-0.16, p=0.001)
hsa-miR-920	1.406	0.583	2.411202	0.016	1.26 (0.52-3.09, p=0.608)	4.08 (1.30-12.78, p=0.016)
(Th-2)						
hsa-miR-101	-1.722	0.699	-2.46331	0.014	0.18 (0.05-0.70, p=0.014)	0.18 (0.05-0.70, p=0.014)
(T-reg)						
hsa-miR-425	0.013	0.004	3.424384	0.001	1.00 (1.00-1.01, p=0.011)	1.01 (1.01-1.02, p=0.001)
hsa-let-7i	-0.02	0.006	-3.299202	0.001	1.00 (0.99-1.00, p=0.390)	0.98 (0.97-0.99, p=0.001)
hsa-miR-16	0.002	0.001	3.203969	0.001	1.00 (1.00-1.00, p=0.046)	1.00 (1.00-1.00, p=0.001)
hsa-let-7g	-0.033	0.015	-2.261216	0.024	0.98 (0.96-1.00, p=0.023)	0.97 (0.94-1.00, p=0.024)
(Stimulatory checkpoint)						
hsa.miR.1202	0.53	0.158	3.350554	0.001	1.13 (1.05-1.21, p=0.001)	1.70 (1.25-2.32, p=0.001)
hsa.miR.101	-7.628	2.62	-2.911913	0.004	0.18 (0.05-0.70, p=0.014)	0.00 (0.00-0.08, p=0.004)
hsa.miR.372	3.333	1.165	2.861809	0.004	0.97 (0.50-1.89, p=0.936)	28.02 (2.86-274.61, p=0.004)
hsa.miR.609	-3.616	1.49	-2.426783	0.015	0.64 (0.19-2.24, p=0.489)	0.03 (0.00-0.50, p=0.015)
(Inhibitory checkpoint)						
hsa.miR.1202	0.53	0.158	3.350554	0.001	1.13 (1.05-1.21, p=0.001)	1.70 (1.25-2.32, p=0.001)
hsa.miR.101	-7.628	2.62	-2.911913	0.004	0.18 (0.05-0.70, p=0.014)	0.00 (0.00-0.08, p=0.004)
hsa.miR.372	3.333	1.165	2.861809	0.004	0.97 (0.50-1.89, p=0.936)	28.02 (2.86-274.61, p=0.004)
hsa.miR.609	-3.616	1.49	-2.426783	0.015	0.64 (0.19-2.24, p=0.489)	0.03 (0.00-0.50, p=0.015)

Note: coef; co-efficiency, se; standard error, HR; hazard ratio, z; z-value.

S5 Table. Significant miRNA candidates in cancer immunity in PCNSL.

	HR[1] (95% CI[2])	P-value	Th-1 Status	Th-2 Status	T-reg Status	Stimulatory Checkpoint	Inhibitory Checkpoint
hsa-miR-1202	1.13 (1.05-1.21)	0.001				●	●
hsa-miR-30d	1.01 (1.00-1.01)	0.002				●	●
hsa-miR-1183	1.71 (1.17-2.50)	0.005				●	●
hsa-miR-425	1.00 (1.00-1.01)	0.011			●		●
hsa-miR-554	0.10 (0.02-0.59)	0.011				●	●
hsa-miR-141	1.14 (1.03-1.27)	0.012	●		●	●	●
hsa-miR-30b	1.01 (1.00-1.01)	0.013				●	●
hsa-miR-875-3p	0.07 (0.01-0.58)	0.013				●	●
hsa-miR-101	0.18 (0.05-0.70)	0.014		●		●	●
hsa-miR-637	1.19 (1.03-1.37)	0.015					●
hsa-miR-422a	1.06 (1.01-1.11)	0.019	●	●		●	●
hsa-miR-577	6.06 (1.32-27.86)	0.021	●	●		●	●
hsa-miR-548d-3p	0.23 (0.07-0.80)	0.021					●
hsa-miR-648	0.19 (0.05-0.79)	0.022				●	●
hsa-let-7g	0.98 (0.96-1.00)	0.023			●	●	
hsa-miR-548b-5p	3.54 (1.19-10.56)	0.024				●	●
hsa-miR-1275	1.01 (1.00-1.03)	0.027	●	●		●	●
hsa-miR-1321	3.06 (1.13-8.34)	0.028	●	●		●	●
hsa-miR-182	1.01 (1.00-1.01)	0.029					●
hsa-miR-1181	1.11 (1.01-1.21)	0.031				●	
hsa-miR-135a	0.27 (0.08-0.92)	0.036		●		●	●
hsa-miR-16	1.00 (1.00-1.00)	0.046	●	●	●	●	●
hsa-miR-649	2.21 (1.01-4.84)	0.046				●	●

Note: [1]HR; hazard ratio, [2]95%CI; 95% confidence interval; sorted by P-value.

S6 Table. Re-evaluated miRNAs with clinical information in PCNSL.

	coef[1]	exp[2](coef)	se[3](coef)	z[4]	p-value	HR[5] (univariable)	HR (multivariable)
**23 miRNAs in training data (n=20)**							
hsa-miR-1202	0.148	1.159	0.062	2.367	0.018	1.13 (1.05-1.21, p=0.001)	1.16 (1.03-1.31)
hsa-miR-101	-3.649	0.026	1.398	-2.610	0.009	0.18 (0.05-0.70, p=0.014)	0.03 (0.00-0.40)
hsa-miR-548b-5p	3.702	40.531	1.264	2.928	0.003	3.54 (1.19-10.56, p=0.024)	40.53 (3.40-482.79)
hsa-miR-554	-3.738	0.024	1.577	-2.370	0.018	0.10 (0.02-0.59, p=0.011)	0.02 (0.00-0.52)
**4 miRNAs + clinical information validation data (n=39)**							
hsa-miR-1202	-0.004	0.996	0.005	-0.840	0.401	1.00 (0.99-1.01, p=0.910)	1.00 (0.99-1.01, p=0.401)
hsa-miR-101	-1.21	0.298	0.652	-1.855	0.064	0.51 (0.26-0.99, p=0.046)	0.30 (0.08-1.07, p=0.064)
hsa-miR-548b-5p	2.132	8.429	0.613	3.478	0.001	1.51 (0.76-3.01, p=0.244)	8.43 (2.54-28.01, p=0.001)
hsa-miR-554	-1.674	0.187	0.69	-2.427	0.015	0.63 (0.32-1.26, p=0.191)	0.19 (0.05-0.72, p=0.015)
age (age>=60, age<60)						0.95 (0.39-2.33, p=0.914)	-
gender (male, female)						1.05 (0.46-2.40, p=0.915)	-
KPS (KPS>=70, KPS<70)						0.37 (0.15-0.92, p=0.033)	-
LDH (LDH>=200, LDH<200)						1.64 (0.71-3.80, p=0.248)	-
deep seated (yes, no)						2.34 (0.94-5.83, p=0.067)	-

Note: [1]coef; co-efficiency, [2]exp; expected value, [3]se; standard error, [4]z; z-value, [5]HR; hazard ratio.

S7 Table. Target candidates of the miRNA predictors for cancer immunity in PCNSL.

Representative miRNA	Target candidates	Gene name	Total context++ score	Th-1 Status	Th-2 Status	T-reg Status	Stimulatory Checkpoint	Inhibitory Checkpoint
hsa-miR-101-3p.1	IL13	interleukin 13	-0.34		IL13			
hsa-miR-101-3p.1	STAT6	signal transducer and activator of transcription 6, interleukin-4 induced	-0.12		STAT6			
hsa-miR-101-3p.1	GATA3	GATA binding protein 3	-0.11		GATA3			
hsa-miR-101-5p	CD96	CD96 molecule	-0.22					CD96
hsa-miR-101-5p	CD40LG	CD40 ligand	-0.19	CD40LG	CD40LG		CD40LG	
hsa-miR-101-5p	TNFSF4	tumor necrosis factor (ligand) superfamily, member 4	-0.14				TNFSF4	
hsa-miR-101-5p	CD274	CD274 molecule	-0.04			CD274		CD274
hsa-miR-101-5p	IL12RB2	interleukin 12 receptor, beta 2	-0.04	IL12RB2				
hsa-miR-101-5p	BTLA	B and T lymphocyte associated	-0.01					BTLA
hsa-miR-1202	TNFRSF14	tumor necrosis factor receptor superfamily, member 14	-0.02				TNFRSF14	TNFRSF14
hsa-miR-1202	CD3D	CD3d molecule, delta (CD3-TCR complex)	-0.01	CD3D	CD3D			
hsa-miR-548a-5p	CD163	CD163 molecule	-0.2			CD163		
hsa-miR-548a-5p	CD3D	CD3d molecule, delta (CD3-TCR complex)	-0.07	CD3D	CD3D			
hsa-miR-548a-5p	STAT1	signal transducer and activator of transcription 1, 91kDa	-0.07	STAT1				
hsa-miR-548a-5p	IL12RB2	interleukin 12 receptor, beta 2	-0.06	IL12RB2				
hsa-miR-548a-5p	CD4	CD4 molecule	-0.02	CD4	CD4	CD4		
hsa-miR-548ab	CD226	CD226 molecule	-0.19				CD226	
hsa-miR-548ab	TGFB3	transforming growth factor, beta 3	-0.09		TGFB3	TGFB3		
hsa-miR-548ab	CD28	CD28 molecule	-0.08	CD28	CD28		CD28	
hsa-miR-548ab	ICOS	inducible T-cell co-stimulator	-0.05				ICOS	
hsa-miR-548ap-5p	CD274	CD274 molecule	-0.19			CD274		CD274
hsa-miR-548ap-5p	BTLA	B and T lymphocyte associated	-0.11					BTLA
hsa-miR-548bb-5p	TNFSF18	tumor necrosis factor (ligand) superfamily, member 18	-0.07				TNFSF18	
hsa-miR-548y	TGFB2	transforming growth factor, beta 2	-0.09			TGFB2		
hsa-miR-554	LTA	lymphotoxin alpha	-0.13	LTA				
hsa-miR-554	CD3D	CD3d molecule, delta (CD3-TCR complex)	-0.12	CD3D	CD3D			
hsa-miR-554	CD28	CD28 molecule	-0.08	CD28	CD28		CD28	
hsa-miR-554	PVR	poliovirus receptor	-0.08				PVR	PVR
hsa-miR-554	CD4	CD4 molecule	-0.07	CD4	CD4	CD4		

Note: TargetScanHuman 7.1/7.2 (http://www.targetscan.org/vert_72/); sorted by Representative miRNA.

S7 Table. Target candidates of the miRNA predictors for cancer immunity in PCNSL.

Representative miRNA	Target candidates	Gene name	Total context++ score	Th-1 Status	Th-2 Status	T-reg Status	Stimulatory Checkpoint	Inhibitory Checkpoint
hsa-miR-101-3p.1	IL13	interleukin 13	-0.34		IL13			
hsa-miR-101-3p.1	STAT6	signal transducer and activator of transcription 6, interleukin-4 induced	-0.12		STAT6			
hsa-miR-101-3p.1	GATA3	GATA binding protein 3	-0.11		GATA3			
hsa-miR-101-5p	CD96	CD96 molecule	-0.22					CD96
hsa-miR-101-5p	CD40LG	CD40 ligand	-0.19	CD40LG	CD40LG		CD40LG	
hsa-miR-101-5p	TNFSF4	tumor necrosis factor (ligand) superfamily, member 4	-0.14				TNFSF4	
hsa-miR-101-5p	CD274	CD274 molecule	-0.04			CD274		CD274
hsa-miR-101-5p	IL12RB2	interleukin 12 receptor, beta 2	-0.04	IL12RB2				
hsa-miR-101-5p	BTLA	B and T lymphocyte associated	-0.01					BTLA
hsa-miR-1202	TNFRSF14	tumor necrosis factor receptor superfamily, member 14	-0.02				TNFRSF14	TNFRSF14
hsa-miR-1202	CD3D	CD3d molecule, delta (CD3-TCR complex)	-0.01	CD3D	CD3D			
hsa-miR-548a-5p	CD163	CD163 molecule	-0.2			CD163		
hsa-miR-548a-5p	CD3D	CD3d molecule, delta (CD3-TCR complex)	-0.07	CD3D	CD3D			
hsa-miR-548a-5p	STAT1	signal transducer and activator of transcription 1, 91kDa	-0.07	STAT1				
hsa-miR-548a-5p	IL12RB2	interleukin 12 receptor, beta 2	-0.06	IL12RB2				
hsa-miR-548a-5p	CD4	CD4 molecule	-0.02	CD4	CD4	CD4		
hsa-miR-548ab	CD226	CD226 molecule	-0.19				CD226	
hsa-miR-548ab	TGFB3	transforming growth factor, beta 3	-0.09		TGFB3	TGFB3		
hsa-miR-548ab	CD28	CD28 molecule	-0.08	CD28	CD28		CD28	
hsa-miR-548ab	ICOS	inducible T-cell co-stimulator	-0.05				ICOS	
hsa-miR-548ap-5p	CD274	CD274 molecule	-0.19			CD274		CD274
hsa-miR-548ap-5p	BTLA	B and T lymphocyte associated	-0.11					BTLA
hsa-miR-548bb-5p	TNFSF18	tumor necrosis factor (ligand) superfamily, member 18	-0.07				TNFSF18	
hsa-miR-548y	TGFB2	transforming growth factor, beta 2	-0.09			TGFB2		
hsa-miR-554	LTA	lymphotoxin alpha	-0.13	LTA				
hsa-miR-554	CD3D	CD3d molecule, delta (CD3-TCR complex)	-0.12	CD3D	CD3D			
hsa-miR-554	CD28	CD28 molecule	-0.08	CD28	CD28		CD28	
hsa-miR-554	PVR	poliovirus receptor	-0.08				PVR	PVR
hsa-miR-554	CD4	CD4 molecule	-0.07	CD4	CD4	CD4		

Note: TargetScanHuman 7.1/7.2 (http://www.targetscan.org/vert_72/); sorted by Representative miRNA.

S8 Table. Characterization of the target candidates of the miRNA predictors for cancer immunity in PCNSL.

Category	Term	Count	%	P-Value
GOTERM_MF_DIRECT	protein binding	495	53.7	3.5E-09
UP_KEYWORDS	Acetylation	212	23	2.0E-07
UP_KEYWORDS	Alternative splicing	539	58.5	3.4E-06
UP_KEYWORDS	Phosphoprotein	430	46.6	1.3E-05
INTERPRO	Small GTPase superfamily, Ras type	10	1.1	5.0E-05
UP_KEYWORDS	Nucleus	285	30.9	6.9E-05
UP_KEYWORDS	Ubl conjugation	110	11.9	7.3E-05
KEGG_PATHWAY	Transcriptional misregulation in cancer	21	2.3	1.0E-04
INTERPRO	Ubiquitin-conjugating enzyme, active site	8	0.9	1.2E-04
UP_KEYWORDS	Prenylation	20	2.2	1.8E-04
INTERPRO	Small GTPase superfamily	18	2	2.0E-04
INTERPRO	Small GTP-binding protein domain	20	2.2	2.2E-04
UP_KEYWORDS	Repressor	46	5	3.5E-04
INTERPRO	Ubiquitin-conjugating enzyme, E2	9	1	5.3E-04
GOTERM_MF_DIRECT	GDP binding	10	1.1	8.7E-04
INTERPRO	Ubiquitin-conjugating enzyme/RWD-like	10	1.1	8.9E-04
UP_KEYWORDS	Cytoplasm	255	27.7	1.1E-03
INTERPRO	Cobalamin (vitamin B12) biosynthesis CobW-like, C-terminal	4	0.4	1.7E-03
INTERPRO	CobW/HypB/UreG domain	4	0.4	1.7E-03
UP_KEYWORDS	Isopeptide bond	72	7.8	2.2E-03
UP_KEYWORDS	Lipoprotein	57	6.2	2.4E-03
BIOCARTA	Signaling of Hepatocyte Growth Factor Receptor	8	0.9	4.3E-03
KEGG_PATHWAY	Pathways in cancer	31	3.4	5.2E-03
INTERPRO	Sas10/Utp3/C1D	3	0.3	6.0E-03
GOTERM_MF_DIRECT	calcium channel regulator activity	6	0.7	7.8E-03
KEGG_PATHWAY	MAPK signaling pathway	22	2.4	8.2E-03
KEGG_PATHWAY	Alcoholism	17	1.8	8.7E-03
KEGG_PATHWAY	Ras signaling pathway	20	2.2	9.5E-03
GOTERM_MF_DIRECT	ubiquitin conjugating enzyme activity	6	0.7	1.1E-02
KEGG_PATHWAY	Protein processing in endoplasmic reticulum	16	1.7	1.3E-02
GOTERM_MF_DIRECT	signal transducer activity	18	2	1.7E-02
KEGG_PATHWAY	Prolactin signaling pathway	9	1	1.7E-02
KEGG_PATHWAY	Chronic myeloid leukemia	9	1	1.9E-02
GOTERM_MF_DIRECT	cyclin-dependent protein serine/threonine kinase activity	6	0.7	2.1E-02
INTERPRO	P-loop containing nucleoside triphosphate hydrolase	54	5.9	2.3E-02
KEGG_PATHWAY	FoxO signaling pathway	13	1.4	2.3E-02
KEGG_PATHWAY	Neurotrophin signaling pathway	12	1.3	2.5E-02
GOTERM_MF_DIRECT	RNA binding	37	4	2.8E-02
GOTERM_MF_DIRECT	GTPase activity	19	2.1	2.9E-02
BIOCARTA	Erk1/Erk2 Mapk Signaling pathway	6	0.7	3.0E-02
GOTERM_MF_DIRECT	single-stranded DNA binding	10	1.1	3.1E-02
GOTERM_MF_DIRECT	ubiquitin protein ligase binding	22	2.4	3.1E-02
BIOCARTA	VEGF, Hypoxia, and Angiogenesis	6	0.7	3.4E-02

Note: DAVID 6.8 (https://david.ncifcrf.gov/); top 10 in each category, P<0.05, sorted by P-value.

S9 Table. Related diseases of the target candidates of the miRNA predictors for cancer immunity in PCNSL.

Category	Term	Count	%	P-Value
GAD_DISEASE	Acquired Immunodeficiency Syndrome|Disease Progression	61	6.6	7.50E-03
GAD_DISEASE	Blood Pressure Determination	13	1.4	1.10E-02
GAD_DISEASE	Fibrinogen	15	1.6	1.30E-02
GAD_DISEASE	Alcoholism	37	4	1.30E-02
GAD_DISEASE	Aorta	8	0.9	1.90E-02
GAD_DISEASE	Blood Coagulation Factors	8	0.9	1.90E-02
GAD_DISEASE	Glucose	20	2.2	2.40E-02
GAD_DISEASE	Body Fat Distribution	11	1.2	2.70E-02
GAD_DISEASE	Abnormalities, Multiple|Heart Defects, Congenital|LEOPARD Syndrome|Noonan Syndrome|Skin Abnormalities	3	0.3	3.10E-02
GAD_DISEASE	Hippocampus	6	0.7	3.10E-02
GAD_DISEASE	Subcutaneous Fat	4	0.4	3.30E-02
GAD_DISEASE	Glioma|Noonan Syndrome|Turner's phenotype, karyotype normal	3	0.3	4.10E-02

Note: DAVID 6.8 (https://david.ncifcrf.gov/); P<0.05, sorted by P-value.

S8 Table. Characterization of the target candidates of the miRNA predictors for cancer immunity in PCNSL.

Category	Term	Count	%	P-Value
GOTERM_MF_DIRECT	protein binding	495	53.7	3.5E-09
UP_KEYWORDS	Acetylation	212	23	2.0E-07
UP_KEYWORDS	Alternative splicing	539	58.5	3.4E-06
UP_KEYWORDS	Phosphoprotein	430	46.6	1.3E-05
INTERPRO	Small GTPase superfamily, Ras type	10	1.1	5.0E-05
UP_KEYWORDS	Nucleus	285	30.9	6.9E-05
UP_KEYWORDS	Ubl conjugation	110	11.9	7.3E-05
KEGG_PATHWAY	Transcriptional misregulation in cancer	21	2.3	1.0E-04
INTERPRO	Ubiquitin-conjugating enzyme, active site	8	0.9	1.2E-04
UP_KEYWORDS	Prenylation	20	2.2	1.8E-04
INTERPRO	Small GTPase superfamily	18	2	2.0E-04
INTERPRO	Small GTP-binding protein domain	20	2.2	2.2E-04
UP_KEYWORDS	Repressor	46	5	3.5E-04
INTERPRO	Ubiquitin-conjugating enzyme, E2	9	1	5.3E-04
GOTERM_MF_DIRECT	GDP binding	10	1.1	8.7E-04
INTERPRO	Ubiquitin-conjugating enzyme/RWD-like	10	1.1	8.9E-04
UP_KEYWORDS	Cytoplasm	255	27.7	1.1E-03
INTERPRO	Cobalamin (vitamin B12) biosynthesis CobW-like, C-terminal	4	0.4	1.7E-03
INTERPRO	CobW/HypB/UreG domain	4	0.4	1.7E-03
UP_KEYWORDS	Isopeptide bond	72	7.8	2.2E-03
UP_KEYWORDS	Lipoprotein	57	6.2	2.4E-03
BIOCARTA	Signaling of Hepatocyte Growth Factor Receptor	8	0.9	4.3E-03
KEGG_PATHWAY	Pathways in cancer	31	3.4	5.2E-03
INTERPRO	Sas10/Utp3/C1D	3	0.3	6.0E-03
GOTERM_MF_DIRECT	calcium channel regulator activity	6	0.7	7.8E-03
KEGG_PATHWAY	MAPK signaling pathway	22	2.4	8.2E-03
KEGG_PATHWAY	Alcoholism	17	1.8	8.7E-03
KEGG_PATHWAY	Ras signaling pathway	20	2.2	9.5E-03
GOTERM_MF_DIRECT	ubiquitin conjugating enzyme activity	6	0.7	1.1E-02
KEGG_PATHWAY	Protein processing in endoplasmic reticulum	16	1.7	1.3E-02
GOTERM_MF_DIRECT	signal transducer activity	18	2	1.7E-02
KEGG_PATHWAY	Prolactin signaling pathway	9	1	1.7E-02
KEGG_PATHWAY	Chronic myeloid leukemia	9	1	1.9E-02
GOTERM_MF_DIRECT	cyclin-dependent protein serine/threonine kinase activity	6	0.7	2.1E-02
INTERPRO	P-loop containing nucleoside triphosphate hydrolase	54	5.9	2.3E-02
KEGG_PATHWAY	FoxO signaling pathway	13	1.4	2.3E-02
KEGG_PATHWAY	Neurotrophin signaling pathway	12	1.3	2.5E-02
GOTERM_MF_DIRECT	RNA binding	37	4	2.8E-02
GOTERM_MF_DIRECT	GTPase activity	19	2.1	2.9E-02
BIOCARTA	Erk1/Erk2 Mapk Signaling pathway	6	0.7	3.0E-02
GOTERM_MF_DIRECT	single-stranded DNA binding	10	1.1	3.1E-02
GOTERM_MF_DIRECT	ubiquitin protein ligase binding	22	2.4	3.1E-02
BIOCARTA	VEGF, Hypoxia, and Angiogenesis	6	0.7	3.4E-02

Note: DAVID 6.8 (https://david.ncifcrf.gov/); top 10 in each category, P<0.05, sorted by P-value.

S9 Table. Related diseases of the target candidates of the miRNA predictors for cancer immunity in PCNSL.

Category	Term	Count	%	P-Value
GAD_DISEASE	Acquired Immunodeficiency Syndrome|Disease Progression	61	6.6	7.50E-03
GAD_DISEASE	Blood Pressure Determination	13	1.4	1.10E-02
GAD_DISEASE	Fibrinogen	15	1.6	1.30E-02
GAD_DISEASE	Alcoholism	37	4	1.30E-02
GAD_DISEASE	Aorta	8	0.9	1.90E-02
GAD_DISEASE	Blood Coagulation Factors	8	0.9	1.90E-02
GAD_DISEASE	Glucose	20	2.2	2.40E-02
GAD_DISEASE	Body Fat Distribution	11	1.2	2.70E-02
GAD_DISEASE	Abnormalities, Multiple|Heart Defects, Congenital|LEOPARD Syndrome|Noonan Syndrome|Skin Abnormalities	3	0.3	3.10E-02
GAD_DISEASE	Hippocampus	6	0.7	3.10E-02
GAD_DISEASE	Subcutaneous Fat	4	0.4	3.30E-02
GAD_DISEASE	Glioma|Noonan Syndrome|Turner's phenotype, karyotype normal	3	0.3	4.10E-02

Note: DAVID 6.8 (https://david.ncifcrf.gov/); P<0.05, sorted by P-value.
