# Supplementary material for: miR-101, miR-548b, miR-554, and miR-1202 are reliable prognosis predictors of the miRNAs associated with cancer immunity in primary central nervous system lymphoma
Source: PLoS One. 2020 Feb 26;15(2):e0229577. doi: 10.1371/journal.pone.0229577 (PMC7043771; doi:10.1371/journal.pone.0229577)
Supplement: S4 Table — (PDF) [file pone.0229577.s008.pdf]

S4 Table. Cox hazard regression analysis for the cancer immunity-related miRNAs in PCNSL.

	coef	se(coef)	z	p-value	HR (univariable)	HR (multivariable)
(Cancer immunity-related miRNAs)						
hsa-miR-1202	0.53	0.158	3.350554	0.001	1.13 (1.05-1.21, p=0.001)	1.70 (1.25-2.32, p=0.001)
hsa-miR-101	-7.628	2.62	-2.911913	0.004	0.18 (0.05-0.70, p=0.014)	0.00 (0.00-0.08, p=0.004)
hsa-miR-372	3.333	1.165	2.861809	0.004	0.97 (0.50-1.89, p=0.936)	28.02 (2.86-274.61, p=0.004)
hsa-miR-609	-3.616	1.49	-2.426783	0.015	0.64 (0.19-2.24, p=0.489)	0.03 (0.00-0.50, p=0.015)
(Th-1)						
hsa-miR-577	6.893	1.848	3.73044	<0.001	6.06 (1.32-27.86, p=0.021)	985.74 (26.35-36870.37, p<0.001)
hsa-miR-454	-4.513	1.373	-3.287874	0.001	0.74 (0.25-2.22, p=0.597)	0.01 (0.00-0.16, p=0.001)
hsa-miR-920	1.406	0.583	2.411202	0.016	1.26 (0.52-3.09, p=0.608)	4.08 (1.30-12.78, p=0.016)
(Th-2)						
hsa-miR-101	-1.722	0.699	-2.46331	0.014	0.18 (0.05-0.70, p=0.014)	0.18 (0.05-0.70, p=0.014)
(T-reg)						
hsa-miR-425	0.013	0.004	3.424384	0.001	1.00 (1.00-1.01, p=0.011)	1.01 (1.01-1.02, p=0.001)
hsa-let-7i	-0.02	0.006	-3.299202	0.001	1.00 (0.99-1.00, p=0.390)	0.98 (0.97-0.99, p=0.001)
hsa-miR-16	0.002	0.001	3.203969	0.001	1.00 (1.00-1.00, p=0.046)	1.00 (1.00-1.00, p=0.001)
hsa-let-7g	-0.033	0.015	-2.261216	0.024	0.98 (0.96-1.00, p=0.023)	0.97 (0.94-1.00, p=0.024)
(Stimulatory checkpoint)						
hsa.miR.1202	0.53	0.158	3.350554	0.001	1.13 (1.05-1.21, p=0.001)	1.70 (1.25-2.32, p=0.001)
hsa.miR.101	-7.628	2.62	-2.911913	0.004	0.18 (0.05-0.70, p=0.014)	0.00 (0.00-0.08, p=0.004)
hsa.miR.372	3.333	1.165	2.861809	0.004	0.97 (0.50-1.89, p=0.936)	28.02 (2.86-274.61, p=0.004)
hsa.miR.609	-3.616	1.49	-2.426783	0.015	0.64 (0.19-2.24, p=0.489)	0.03 (0.00-0.50, p=0.015)
(Inhibitory checkpoint)						
hsa.miR.1202	0.53	0.158	3.350554	0.001	1.13 (1.05-1.21, p=0.001)	1.70 (1.25-2.32, p=0.001)
hsa.miR.101	-7.628	2.62	-2.911913	0.004	0.18 (0.05-0.70, p=0.014)	0.00 (0.00-0.08, p=0.004)
hsa.miR.372	3.333	1.165	2.861809	0.004	0.97 (0.50-1.89, p=0.936)	28.02 (2.86-274.61, p=0.004)
hsa.miR.609	-3.616	1.49	-2.426783	0.015	0.64 (0.19-2.24, p=0.489)	0.03 (0.00-0.50, p=0.015)

Note: coef; co-efficiency, se; standard error, HR; hazard ratio, z; z-value.
