# Supplementary material for: miR-101, miR-548b, miR-554, and miR-1202 are reliable prognosis predictors of the miRNAs associated with cancer immunity in primary central nervous system lymphoma
Source: PLoS One. 2020 Feb 26;15(2):e0229577. doi: 10.1371/journal.pone.0229577 (PMC7043771; doi:10.1371/journal.pone.0229577)
Supplement: S5 Table — (PDF) [file pone.0229577.s009.pdf]

S5 Table. Significant miRNA candidates in cancer immunity in PCNSL.

	HR[1] (95% CI[2])	P-value	Th-1 Status	Th-2 Status	T-reg Status	Stimulatory Checkpoint	Inhibitory Checkpoint
hsa-miR-1202	1.13 (1.05-1.21)	0.001				●	●
hsa-miR-30d	1.01 (1.00-1.01)	0.002				●	●
hsa-miR-1183	1.71 (1.17-2.50)	0.005				●	●
hsa-miR-425	1.00 (1.00-1.01)	0.011			●		●
hsa-miR-554	0.10 (0.02-0.59)	0.011				●	●
hsa-miR-141	1.14 (1.03-1.27)	0.012	●		●	●	●
hsa-miR-30b	1.01 (1.00-1.01)	0.013				●	●
hsa-miR-875-3p	0.07 (0.01-0.58)	0.013				●	●
hsa-miR-101	0.18 (0.05-0.70)	0.014		●		●	●
hsa-miR-637	1.19 (1.03-1.37)	0.015					●
hsa-miR-422a	1.06 (1.01-1.11)	0.019	●	●		●	●
hsa-miR-577	6.06 (1.32-27.86)	0.021	●	●		●	●
hsa-miR-548d-3p	0.23 (0.07-0.80)	0.021					●
hsa-miR-648	0.19 (0.05-0.79)	0.022				●	●
hsa-let-7g	0.98 (0.96-1.00)	0.023			●	●	
hsa-miR-548b-5p	3.54 (1.19-10.56)	0.024				●	●
hsa-miR-1275	1.01 (1.00-1.03)	0.027	●	●		●	●
hsa-miR-1321	3.06 (1.13-8.34)	0.028	●	●		●	●
hsa-miR-182	1.01 (1.00-1.01)	0.029					●
hsa-miR-1181	1.11 (1.01-1.21)	0.031				●	
hsa-miR-135a	0.27 (0.08-0.92)	0.036		●		●	●
hsa-miR-16	1.00 (1.00-1.00)	0.046	●	●	●	●	●
hsa-miR-649	2.21 (1.01-4.84)	0.046				●	●

Note: [1]HR; hazard ratio, [2]95%CI; 95% confidence interval; sorted by P-value.
