# Supplementary material for: miR-101, miR-548b, miR-554, and miR-1202 are reliable prognosis predictors of the miRNAs associated with cancer immunity in primary central nervous system lymphoma
Source: PLoS One. 2020 Feb 26;15(2):e0229577. doi: 10.1371/journal.pone.0229577 (PMC7043771; doi:10.1371/journal.pone.0229577)
Supplement: S6 Table — (PDF) [file pone.0229577.s010.pdf]

S6 Table. Re-evaluated miRNAs with clinical information in PCNSL.

	coef[1]	exp[2](coef)	se[3](coef)	z[4]	p-value	HR[5] (univariable)	HR (multivariable)
**23 miRNAs in training data (n=20)**							
hsa-miR-1202	0.148	1.159	0.062	2.367	0.018	1.13 (1.05-1.21, p=0.001)	1.16 (1.03-1.31)
hsa-miR-101	-3.649	0.026	1.398	-2.610	0.009	0.18 (0.05-0.70, p=0.014)	0.03 (0.00-0.40)
hsa-miR-548b-5p	3.702	40.531	1.264	2.928	0.003	3.54 (1.19-10.56, p=0.024)	40.53 (3.40-482.79)
hsa-miR-554	-3.738	0.024	1.577	-2.370	0.018	0.10 (0.02-0.59, p=0.011)	0.02 (0.00-0.52)
**4 miRNAs + clinical information validation data (n=39)**							
hsa-miR-1202	-0.004	0.996	0.005	-0.840	0.401	1.00 (0.99-1.01, p=0.910)	1.00 (0.99-1.01, p=0.401)
hsa-miR-101	-1.21	0.298	0.652	-1.855	0.064	0.51 (0.26-0.99, p=0.046)	0.30 (0.08-1.07, p=0.064)
hsa-miR-548b-5p	2.132	8.429	0.613	3.478	0.001	1.51 (0.76-3.01, p=0.244)	8.43 (2.54-28.01, p=0.001)
hsa-miR-554	-1.674	0.187	0.69	-2.427	0.015	0.63 (0.32-1.26, p=0.191)	0.19 (0.05-0.72, p=0.015)
age (age>=60, age<60)						0.95 (0.39-2.33, p=0.914)	-
gender (male, female)						1.05 (0.46-2.40, p=0.915)	-
KPS (KPS>=70, KPS<70)						0.37 (0.15-0.92, p=0.033)	-
LDH (LDH>=200, LDH<200)						1.64 (0.71-3.80, p=0.248)	-
deep seated (yes, no)						2.34 (0.94-5.83, p=0.067)	-

Note: [1]coef; co-efficiency, [2]exp; expected value, [3]se; standard error, [4]z; z-value, [5]HR; hazard ratio.
