# Supplementary material for: miR-101, miR-548b, miR-554, and miR-1202 are reliable prognosis predictors of the miRNAs associated with cancer immunity in primary central nervous system lymphoma
Source: PLoS One. 2020 Feb 26;15(2):e0229577. doi: 10.1371/journal.pone.0229577 (PMC7043771; doi:10.1371/journal.pone.0229577)
Supplement: S7 Table — (PDF) [file pone.0229577.s011.pdf]

S7 Table. Target candidates of the miRNA predictors for cancer immunity in PCNSL.

Representative miRNA	Target candidates	Gene name	Total context++ score	Th-1 Status	Th-2 Status	T-reg Status	Stimulatory Checkpoint	Inhibitory Checkpoint
hsa-miR-101-3p.1	IL13	interleukin 13	-0.34		IL13			
hsa-miR-101-3p.1	STAT6	signal transducer and activator of transcription 6, interleukin-4 induced	-0.12		STAT6			
hsa-miR-101-3p.1	GATA3	GATA binding protein 3	-0.11		GATA3			
hsa-miR-101-5p	CD96	CD96 molecule	-0.22					CD96
hsa-miR-101-5p	CD40LG	CD40 ligand	-0.19	CD40LG	CD40LG		CD40LG	
hsa-miR-101-5p	TNFSF4	tumor necrosis factor (ligand) superfamily, member 4	-0.14				TNFSF4	
hsa-miR-101-5p	CD274	CD274 molecule	-0.04			CD274		CD274
hsa-miR-101-5p	IL12RB2	interleukin 12 receptor, beta 2	-0.04	IL12RB2				
hsa-miR-101-5p	BTLA	B and T lymphocyte associated	-0.01					BTLA
hsa-miR-1202	TNFRSF14	tumor necrosis factor receptor superfamily, member 14	-0.02				TNFRSF14	TNFRSF14
hsa-miR-1202	CD3D	CD3d molecule, delta (CD3-TCR complex)	-0.01	CD3D	CD3D			
hsa-miR-548a-5p	CD163	CD163 molecule	-0.2			CD163		
hsa-miR-548a-5p	CD3D	CD3d molecule, delta (CD3-TCR complex)	-0.07	CD3D	CD3D			
hsa-miR-548a-5p	STAT1	signal transducer and activator of transcription 1, 91kDa	-0.07	STAT1				
hsa-miR-548a-5p	IL12RB2	interleukin 12 receptor, beta 2	-0.06	IL12RB2				
hsa-miR-548a-5p	CD4	CD4 molecule	-0.02	CD4	CD4	CD4		
hsa-miR-548ab	CD226	CD226 molecule	-0.19				CD226	
hsa-miR-548ab	TGFB3	transforming growth factor, beta 3	-0.09		TGFB3	TGFB3		
hsa-miR-548ab	CD28	CD28 molecule	-0.08	CD28	CD28		CD28	
hsa-miR-548ab	ICOS	inducible T-cell co-stimulator	-0.05				ICOS	
hsa-miR-548ap-5p	CD274	CD274 molecule	-0.19			CD274		CD274
hsa-miR-548ap-5p	BTLA	B and T lymphocyte associated	-0.11					BTLA
hsa-miR-548bb-5p	TNFSF18	tumor necrosis factor (ligand) superfamily, member 18	-0.07				TNFSF18	
hsa-miR-548y	TGFB2	transforming growth factor, beta 2	-0.09			TGFB2		
hsa-miR-554	LTA	lymphotoxin alpha	-0.13	LTA				
hsa-miR-554	CD3D	CD3d molecule, delta (CD3-TCR complex)	-0.12	CD3D	CD3D			
hsa-miR-554	CD28	CD28 molecule	-0.08	CD28	CD28		CD28	
hsa-miR-554	PVR	poliovirus receptor	-0.08				PVR	PVR
hsa-miR-554	CD4	CD4 molecule	-0.07	CD4	CD4	CD4		

Note: TargetScanHuman 7.1/7.2 (http://www.targetscan.org/vert_72/); sorted by Representative miRNA.
