# Supplementary material for: miR-101, miR-548b, miR-554, and miR-1202 are reliable prognosis predictors of the miRNAs associated with cancer immunity in primary central nervous system lymphoma
Source: PLoS One. 2020 Feb 26;15(2):e0229577. doi: 10.1371/journal.pone.0229577 (PMC7043771; doi:10.1371/journal.pone.0229577)
Supplement: S8 Table — (PDF) [file pone.0229577.s012.pdf]

S8 Table. Characterization of the target candidates of the miRNA predictors for cancer immunity in PCNSL.

Category	Term	Count	%	P-Value
GOTERM_MF_DIRECT	protein binding	495	53.7	3.5E-09
UP_KEYWORDS	Acetylation	212	23	2.0E-07
UP_KEYWORDS	Alternative splicing	539	58.5	3.4E-06
UP_KEYWORDS	Phosphoprotein	430	46.6	1.3E-05
INTERPRO	Small GTPase superfamily, Ras type	10	1.1	5.0E-05
UP_KEYWORDS	Nucleus	285	30.9	6.9E-05
UP_KEYWORDS	Ubl conjugation	110	11.9	7.3E-05
KEGG_PATHWAY	Transcriptional misregulation in cancer	21	2.3	1.0E-04
INTERPRO	Ubiquitin-conjugating enzyme, active site	8	0.9	1.2E-04
UP_KEYWORDS	Prenylation	20	2.2	1.8E-04
INTERPRO	Small GTPase superfamily	18	2	2.0E-04
INTERPRO	Small GTP-binding protein domain	20	2.2	2.2E-04
UP_KEYWORDS	Repressor	46	5	3.5E-04
INTERPRO	Ubiquitin-conjugating enzyme, E2	9	1	5.3E-04
GOTERM_MF_DIRECT	GDP binding	10	1.1	8.7E-04
INTERPRO	Ubiquitin-conjugating enzyme/RWD-like	10	1.1	8.9E-04
UP_KEYWORDS	Cytoplasm	255	27.7	1.1E-03
INTERPRO	Cobalamin (vitamin B12) biosynthesis CobW-like, C-terminal	4	0.4	1.7E-03
INTERPRO	CobW/HypB/UreG domain	4	0.4	1.7E-03
UP_KEYWORDS	Isopeptide bond	72	7.8	2.2E-03
UP_KEYWORDS	Lipoprotein	57	6.2	2.4E-03
BIOCARTA	Signaling of Hepatocyte Growth Factor Receptor	8	0.9	4.3E-03
KEGG_PATHWAY	Pathways in cancer	31	3.4	5.2E-03
INTERPRO	Sas10/Utp3/C1D	3	0.3	6.0E-03
GOTERM_MF_DIRECT	calcium channel regulator activity	6	0.7	7.8E-03
KEGG_PATHWAY	MAPK signaling pathway	22	2.4	8.2E-03
KEGG_PATHWAY	Alcoholism	17	1.8	8.7E-03
KEGG_PATHWAY	Ras signaling pathway	20	2.2	9.5E-03
GOTERM_MF_DIRECT	ubiquitin conjugating enzyme activity	6	0.7	1.1E-02
KEGG_PATHWAY	Protein processing in endoplasmic reticulum	16	1.7	1.3E-02
GOTERM_MF_DIRECT	signal transducer activity	18	2	1.7E-02
KEGG_PATHWAY	Prolactin signaling pathway	9	1	1.7E-02
KEGG_PATHWAY	Chronic myeloid leukemia	9	1	1.9E-02
GOTERM_MF_DIRECT	cyclin-dependent protein serine/threonine kinase activity	6	0.7	2.1E-02
INTERPRO	P-loop containing nucleoside triphosphate hydrolase	54	5.9	2.3E-02
KEGG_PATHWAY	FoxO signaling pathway	13	1.4	2.3E-02
KEGG_PATHWAY	Neurotrophin signaling pathway	12	1.3	2.5E-02
GOTERM_MF_DIRECT	RNA binding	37	4	2.8E-02
GOTERM_MF_DIRECT	GTPase activity	19	2.1	2.9E-02
BIOCARTA	Erk1/Erk2 Mapk Signaling pathway	6	0.7	3.0E-02
GOTERM_MF_DIRECT	single-stranded DNA binding	10	1.1	3.1E-02
GOTERM_MF_DIRECT	ubiquitin protein ligase binding	22	2.4	3.1E-02
BIOCARTA	VEGF, Hypoxia, and Angiogenesis	6	0.7	3.4E-02

Note: DAVID 6.8 (https://david.ncifcrf.gov/); top 10 in each category, P<0.05, sorted by P-value.
