# Supplementary material for: miR-101, miR-548b, miR-554, and miR-1202 are reliable prognosis predictors of the miRNAs associated with cancer immunity in primary central nervous system lymphoma
Source: PLoS One. 2020 Feb 26;15(2):e0229577. doi: 10.1371/journal.pone.0229577 (PMC7043771; doi:10.1371/journal.pone.0229577)
Supplement: S9 Table — (PDF) [file pone.0229577.s013.pdf]

S9 Table. Related diseases of the target candidates of the miRNA predictors for cancer immunity in PCNSL.

Category	Term	Count	%	P-Value
GAD_DISEASE	Acquired Immunodeficiency Syndrome|Disease Progression	61	6.6	7.50E-03
GAD_DISEASE	Blood Pressure Determination	13	1.4	1.10E-02
GAD_DISEASE	Fibrinogen	15	1.6	1.30E-02
GAD_DISEASE	Alcoholism	37	4	1.30E-02
GAD_DISEASE	Aorta	8	0.9	1.90E-02
GAD_DISEASE	Blood Coagulation Factors	8	0.9	1.90E-02
GAD_DISEASE	Glucose	20	2.2	2.40E-02
GAD_DISEASE	Body Fat Distribution	11	1.2	2.70E-02
GAD_DISEASE	Abnormalities, Multiple|Heart Defects, Congenital|LEOPARD Syndrome|Noonan Syndrome|Skin Abnormalities	3	0.3	3.10E-02
GAD_DISEASE	Hippocampus	6	0.7	3.10E-02
GAD_DISEASE	Subcutaneous Fat	4	0.4	3.30E-02
GAD_DISEASE	Glioma|Noonan Syndrome|Turner's phenotype, karyotype normal	3	0.3	4.10E-02

Note: DAVID 6.8 (https://david.ncifcrf.gov/); P<0.05, sorted by P-value.
